# A Computational Analysis of Crystallite Shape under Quiescent and Stretch-Induced Polyethylene Crystallization

**DOI:** 10.1021/acs.macromol.5c02153

**Published:** 2025-11-30

**Authors:** Fotis Venetsanos, Stefanos D. Anogiannakis, Doros N. Theodorou

**Affiliations:** School of Chemical Engineering, 150569 National Technical University of Athens, 9 Heroon Polytechniou Street, 15780 Athens, Greece

## Abstract

One of the most remarkable properties of crystallizable polymers is their ability to form complex semicrystalline morphologies, which are responsible for their excellent barrier and very good mechanical properties. These morphologies are sensitive to various processing and material-dependent factors such as the temperature, the flow type and strain rate, the molecular architecture and the size distribution of polymer chains; their prediction is challenging. Atomistic simulations can offer unique insight into the mechanisms that govern polymer crystallization, elucidating aspects of nucleation and growth under different processing conditions. In this work, starting from Monte Carlo equilibrated linear polyethylene melts of a uniform molecular weight distribution, we perform isothermal molecular dynamics simulations of crystallization under two different protocols, i.e., under (a) quiescent and (b) stretching conditions. Comparing between the two protocols, we quantify how the presence of a flow field affects the emerging semicrystalline morphology. Using home-built algorithms, we calculate the evolution of the degree of crystallinity over time, analyze the mass and the radius of gyration tensor of the largest ordered cluster present, and determine the stochastic distribution of induction times from the aforementioned geometric characteristics and from mean first passage time analysis. We show that the presence of a flow field has strong impact on nucleation and growth, accelerating the emergence of the crystalline phase. We establish a methodology for quantifying the shape and orientation of the emerging crystallites and of their constituent atoms through a radius of gyration tensor and **Q**-tensor analysis and highlight differences in their evolution between the two different crystallization protocols. Crystallites created under stretching are strongly oriented along the drawing direction and ultimately adopt a more cylindrical symmetry, as opposed to the quasi-spherical clusters generated under quiescent conditions, whose orientation is random. We quantify—via the eigenvalues of the diagonalized radius of gyration tensor—the growth rate along the principal axes of the crystallites, as well as their volumetric growth rate. Finally, we investigate our quiescent specimens at long times, presenting evidence of semicrystalline morphologies such as bridging between different crystallites and curving of the lamellar planes. We showcase, throughout our study, links between crystallite morphology and the stages of nucleation and growth, shedding additional light on the mechanisms which govern polymer crystallization. All of our findings are accompanied by three-dimensional visualizations of the systems under study, illuminating the parameters and mechanisms analyzed, as well as the influence of stretching on the orientation of the chains participating in the crystalline phase. We validate the various parameters and measures obtained through our different methods of analysis against each other.

## Graphical abstract

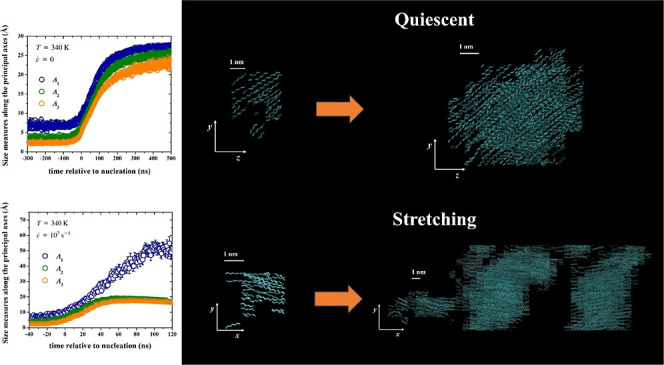

## 1 Introduction

The ability of crystallizable polymers to form complex semicrystalline structures is a key factor for their excellent barrier^1^ and very good mechanical properties.^2^ Studying the nascence of the crystalline phase, determining and quantifying the mechanisms which take place and monitoring the evolution of shape, size, and orientation of ordered clusters in the emerging structure is of utmost importance for the design of new materials, as it can provide direct links between process conditions, molecular characteristics, morphology, and macroscopic properties. Employing atomistic simulations enables a deep exploration of the microstructure to uncover the mechanisms which govern nucleation and growth.

Polymer crystallization, and polyethylene (PE) crystallization, in particular, is being studied extensively. It has developed into a focal point for molecular simulations and materials science, notably in the domain of eco-friendly recyclable packaging materials. Today there is a need to redesign packaging plastics, one of the world’s leading sources of pollution,^3,4^ within the principles of circular economy.^5,6^

Extensive experimental studies have been able to identify a plethora of properties of semicrystalline polyethylene produced under different processing standards, which include both quiescent^1,2,7,13,14^ and stretching^15–17^ conditions. Measurements of strength^2,7,8^ and permeability^1,9,10^ are essential for the characterization of the final product. Experimentalists are also able to quantify crystal growth,^11,12^ including the shish-kebab morphology developed under stretching,^17^ and to measure the lamellar thickness^11,13^ in early, microscopic domains, via small angle neutron scattering (SANS) and differential scanning calorimetry (DSC), as well as to quantify the more macroscopic spherulitic growth through optical and microscopic techniques.

Molecular simulations play an integral part in identifying and characterizing in depth the mechanisms that drive polyethylene crystallization. The evolution of simulation studies has been impressive, starting from modest, oligomer or single chain systems,^18–24^ continuing with longer, entangled chains,^25–35^ moving to “heroic” state-of-the-art simulations of outstandingly large systems,^29,32^ and branching out to the development of predictive mesoscopic models.^36–40^ Various properties have been investigated, including characteristic parameters of crystallization kinetics such as the nucleation and growth rates^25,33^ and their connections to the morphology,^33^ gas solubility^34^ and strain history^32^ of the systems.

As with every fertile scientific field, however, there are many areas that still remain uncovered. In this work we aim to fulfill two objectives: (a) to shed light on the kinetics that govern polyethylene crystallization, and (b) to define in detail the shape and orientation of emerging crystallites and their evolution under quiescent conditions and under uniaxial stretching. With these objectives in mind, we perform large-scale molecular dynamics (MD) simulations on entangled linear PE systems with a narrow, uniform molecular weight distribution. In contrast to previous computational works, the starting melt systems used here have undergone thorough equilibration through our home-built connectivity-altering Monte Carlo (MC) algorithm.^41^ Thanks to the MC, a broad sampling of 24 different uncorrelated initial configurations becomes possible, which is extremely beneficial in studying the rare event nature of crystallization in the subsequent long MD simulations.

We conduct our studies at significantly deeper subcooling compared to experimental and process standards. This is because MD is very limited in terms of time scale (in most cases being able to simulate decades of μs on commonly available computational platforms), while real-life crystallization process times are several orders of magnitude longer, typically ranging from seconds to minutes.^14,17^ Crystallization under quiescent conditions is the main limiting case, as much longer times are required in comparison to stretch-induced crystallization. A temperature that allows observing quiescent crystallization of PE at a reasonable rate with MD is *T* = 340 K; it is the temperature used throughout this study. We are able to crystallize similar PE systems under stretching at higher temperatures;^33^ here, however, we focus on comparing crystallization in the absence or presence of an external flow field, and consistency demands that both cases be simulated at the same temperature, as crystallization is very sensitive to temperature changes.

To investigate the kinetics of crystallization we employ the Mean First-Passage Time (MFPT) methodology,^25,42^ used also in our previous study of PE crystallization under plane strain^33^ and other works focusing on the kinetics of polymer crystallization.^23,25,43,44^ MFPT serves as an excellent scale, against which we can corroborate the results extracted from the significant novel methodologies presented in our work. In addition, we compute a cumulative probability distribution of the induction times, individually measured from our specimens through a new graphical methodology based on tracking the radius of gyration of the largest crystalline cluster of each single trajectory as a function of time. Acquiring enough data to feed a cumulative probability distribution model is an original accomplishment for the field of molecular dynamics and has been enabled by our homebrewed connectivity-altering MC algorithm. Estimates from this new methodology are compared against MFPT estimates and results are found to be consistent. The evolution of crystallite size, shape and orientation, is addressed through an implementation of a radius of gyration tensor^45^ analysis. Furthermore, original insight is offered as the local orientation of chain stems constituting the ordered clusters is quantified through **Q**-tensor analysis.^46^ By diagonalizing the radius of gyration tensor and the **Q**-tensor one is able to extract shape and orientation measures for the cluster as a whole body and for the chains of which it consists. Shape is quantified through the mean squared radius of gyration, asphericity and acylindricity, while orientation through a scalar order parameter. The square roots of the eigenvalues of the radius of gyration tensor serve as measures of the lengths of the principal axes. These are studied as functions of time, leading to measures of the local (anisotropic) growth rate. Elements of the radius of gyration and the **Q**-tensor have been used in the past in a rigorous analysis of biaxial stretch-induced crystallization of coarse-grained poly­(vinyl alcohol) (CG-PVA) through molecular dynamics simulations.^47^ In that work, semicrystalline specimens of CG-PVA were produced by creating oriented configurations in the melt, quenching the systems to temperatures below the melting point under a variety of biaxial strain conditions, and observing nucleation and growth. An important contribution of ref 47 was the quantification of global and local orientation parameters, as well as of crystal growth along the thickness, lateral and *z*-axis directions. In addition, ref 47 elaborated on crystal structure and morphology, pointing out the merging of crystalline clusters in highly oriented melts, and proposing it as a way to form shish morphologies. Our methodology aims to use these measures to gain new insight by adhering to the following, important aspects: (a) the introduction of Monte Carlo equilibrated, statistically independent initial configurations, (b) the emergence of crystalline morphology occurring without preordering in the melt phase, (c) a systematic study of quiescent crystallization without constraints, aside from periodic boundary conditions, (d) quantifying not only the size, but also the shape evolution of the emerging crystallites as a function of time, (e) correlating the shape and orientation of the crystallites relative to each other, as well as relative to the orientation of the chords constituting the crystalline strands, and explicitly linking the geometric information extracted to the observed nucleation and growth rates. The consideration of all of the above aims to pave a robust way of investigating the evolution of the microscopic morphology of a PE crystal nucleus during nucleation and growth.

It is important to clarify that here we choose to keep our postprocessing analysis as simple as possible by focusing solely on the largest cluster and using its size as our reaction coordinate. This allows us to directly calculate the nucleation and growth rate, as well as the above-mentioned shape evolution parameters, and make comparisons against MFPT. In doing so, however, we deemphasize aspects involving multiple clusters, especially under stretching, such as determining the cluster merging and demerging rates, and evaluating the possible contribution of smaller crystals to the overall crystallization behavior. An expansion of our methodology to an analysis of the number of crystals, their size distribution and other relevant metrics could provide a more complete picture of the crystallization process under both quiescent and stretching conditions.

The rest of this article is structured as follows. In Section 2 we describe the generation of the PE systems used in our simulation, the force field, and the MD simulation parameters. In Section 3 we describe the methodologies employed to achieve our objectives, namely our simulation protocol, the postprocessing method used for determining the crystalline regions, measuring the degree of crystallinity and performing a cluster analysis. Furthermore, we present our shape and orientation analysis, explaining how we diagonalize (a) the radius of gyration tensor and (b) the **Q**-tensor and how we calculate key quantities such as (i) the mean squared radius of gyration, reduced asphericity and reduced acylindricity, and (ii) the scalar order parameter and the director of chords in a crystalline cluster. We also explain how we quantify the directional correlation of the longest principal axis with each one of the axes of the laboratory frame and with the local director. In Section 4 we present and discuss our findings, accompanied by visualizations of our systems through three-dimensional (3D) representations. We also compare the parameters and measures obtained from our different methodologies against each other. We have to note that our simulations have been conducted under temperature and stretch rate conditions that have not yet been studied experimentally. Our computational findings provide essential insight but cannot be validated directly against experiments, as the quantities measured are currently beyond the limits of experimental characterization.

In Section 5 we summarize the most important conclusions from this work. The findings from our study have great potential, as monitoring the shape and chain orientation evolution of emerging domains of the crystalline phase and quantifying the growth rate in different directions can serve as basic ingredients for moving to a mesoscopic model for polymer crystallization, able to address length and time scales encountered in common industrial processes.

## 2 Systems Studied

In this work we simulated 24 different, Monte Carlo (MC) equilibrated, linear polyethylene melt systems. Each system consisted of 100 chains, with an average length of 1000 methylene units and dispersity *D̵*= 1.08, corresponding to a uniform number distribution of molar masses ranging from ${\mathrm{M̅}}_{n}$ /2 to 3 ${\mathrm{M̅}}_{n}$ /2 with ${\mathrm{M̅}}_{n}$ being the number-average molar mass. This molar mass distribution is identical across all 24 simulated systems. It has been introduced in order to enable full equilibration of our melt systems with our connectivity-altering Monte Carlo algorithm. Achieving this equilibration is absolutely essential for getting meaningful results, given the infrequent event nature of crystal nucleation. A dispersity of 1.08 was chosen purposefully, as it will not produce results substantially different from a monodisperse simulation. Effects introduced by large polydispersities to the mechanism, rate, and morphology is certainly a problem we want to take up and study systematically in the future, in view of the importance of dispersity in monomaterial packaging. The TraPPE-UA force field^48^ was employed during both the generation and MD simulations of all systems. In this force field, all PE chains are represented as sequences of united atoms (UAs), corresponding to the skeletal carbon atoms onto which the pendant hydrogens are collapsed. The application of such a force field affords significant reduction of computation time relative to fully atomistic representations, and this is crucial for the elaborate MD simulations performed in this work.

TraPPE-UA and similar UA force fields have been shown to describe PE in the melt accurately,^49,50^ reproducing several thermodynamic, structural, conformational, rheological and dynamical^51^ properties very well. Although such UA force fields lack the ability to predict the exact orthorhombic structure of crystalline PE, they have been used extensively in studies of semicrystalline PE, yielding very reasonable results,^31–34^ including an estimation of gas solubility in semicrystalline PE.^34^

A melt configuration was initially generated at *T* = 450 K using the amorphous builder plugin, courtesy of the MAPS platform developed by Scienomics,^52^ in a cubic simulation box, where periodic boundary conditions were applied. It was then subjected to a short (20 ns) *NVT* MD simulation, followed by another short (20 ns) *NpT* MD simulation, using GROMACS,^53–55^ for chain relaxation and density equilibration, respectively. The final configuration obtained was then used as an input to our home-built connectivity-altering MC algorithm,^41^ at constant temperature, *T* = 450 K, and pressure, *p* = 1 atm, resulting in a trajectory of 10 billion MC steps, conforming to a uniform number distribution of molecular weights, with *D̵*= 1.08. We sampled this MC trajectory, extracting 24 equilibrated and statistically independent PE melt configurations. The root-mean-square radius of gyration of the melt systems at 450 K is ⟨*R*_g_^2^⟩ = 54.0 Å, the average density of the systems is ρ = 0.77 g/cm^3^ and the average cubic simulation box length is *L*_0_ = 144.0 Å. We measured Flory’s characteristic ratio at *C*_∞_ = 8.5, in good accordance with past simulation^56,57^ and experimental data.^58^ The slightly elevated characteristic ratio value can be attributed to the TraPPe-UA force field, which favors the all-trans configuration.^59^

Our MD simulations of crystallization are described in detail in the Methodology section; here we provide only a very coarse outline, to orient the reader. We study our PE specimens under two distinct protocols: crystallization under quiescent conditions and crystallization under stretching. Initially, in both cases we cool down our melt samples to 340 K, applying a constant, relatively fast cooling rate of 10 K/ns in order to avoid the development of crystallinity during this initial cooling process.

Under quiescent conditions of temperature *T* = 340 K and pressure *p* = 1 atm, each specimen is left to relax in the *NpT* ensemble for several hundreds of nanoseconds, the total time depending on how fast nucleation occurs. In the course of the MD simulation, we monitor the degree of crystallinity via our home-built postprocessing algorithm, described in the methodology section. We terminate our simulations when each specimen reaches a 30% degree of crystallinity.

For stretching we apply an external, uniaxial flow field to our cooled, initially amorphous structures, performing isothermal (*T* = 340 K) MD simulations under constant strain rate ε̇ = 10^7^ s^–1^ in the *x*-direction, with the *y* and *z* directions exposed to an ambient pressure of *p* = 1 atm. The simulation time was the same across all stretching simulations, 150 ns, and the final draw ratio was equal to 2.5.

## 3 Methodology

### 3.1 Simulation Protocol

In this work we focus on two polyethylene crystallization scenarios. Crystallization under quiescent conditions, and stretch-induced crystallization. The initial stage is shared in both cases. Starting from the melt, at 450 K, employing a cooling rate of 10 K/ns, we cool our systems down to a target temperature of 340 K. There we allow them to relax under quiescent conditions, at constant temperature *T* = 340 K and pressure *p* = 1 atm, performing MD simulations in the *NpT* ensemble. Periodic boundary conditions are applied in all three directions at all times. This temperature is well below the melting point of PE, and was chosen as we expect to get induction times that are short enough to observe within a MD run (a few hundred nanoseconds). The simulation time varies; homogeneous nucleation being an infrequent event, there is a very broad range of potential induction times. In order to perform a comprehensive analysis of the metrics obtained and to be able to accumulate averaged quantities along our trajectories, the terminal point of each simulation was chosen to be at a degree of crystallinity *x*_c_ = 0.3. That is slightly before the time-dependent *x*_c_ value reaches a plateau, but it was imperative to minimize the computational cost of our simulations. Also, the phenomena of interest in this work, crystal nucleation and growth, are captured excellently within the simulated time frame. In Figure 1 we display *xy* projections of a system under quiescent crystallization, in the initial, fully amorphous phase, and after crystalline regions have developed. Pink and cyan chain segments correspond to the amorphous and crystalline domains of the system, respectively, as identified by the method we will describe in the following subsection.

**Figure 1:**
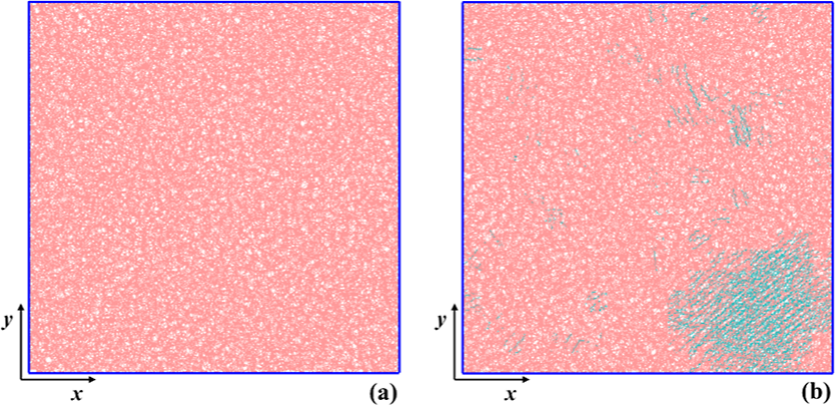
(a) Initial purely amorphous PE configuration, (b) the same system postnucleation. Amorphous and crystalline domains are denoted with pink and cyan colors, respectively. The *xy*-projections were created using the VMD software.^60,61^

In order to be able to compare between our two cases, stretch-induced crystallization is also performed at 340 K, initially cooling the melt down at a rate of 10 K/ns and allowing for a very short density equilibration of 10 ns. We then apply a uniaxial extensional flow field, performing MD simulations under constant engineering strain rate, ε̇, equal to 10^7^ s^–1^ in the *x* direction, while the *y* and *z* directions are exposed to an ambient pressure of 1 atm. The temperature remains constant at *T* = 340 K. During this transient process, the specimen thickness is reduced along the *y* and *z* axes as drawing progresses. Periodic boundary conditions are applied in all three directions at all times. The duration of each drawing simulation is 150 ns, and the final draw ratio is 2.5. In Figure 2 we display *xy* projections of a system under stretch-induced crystallization, in the initial, undeformed, fully amorphous phase (λ = 1), and in a deformed state (λ = 1.75), in which crystalline regions have developed. Pink and cyan chain segments correspond to the amorphous and crystalline domains of the system, respectively, as identified by the method we describe in the following subsection.

**Figure 2:**
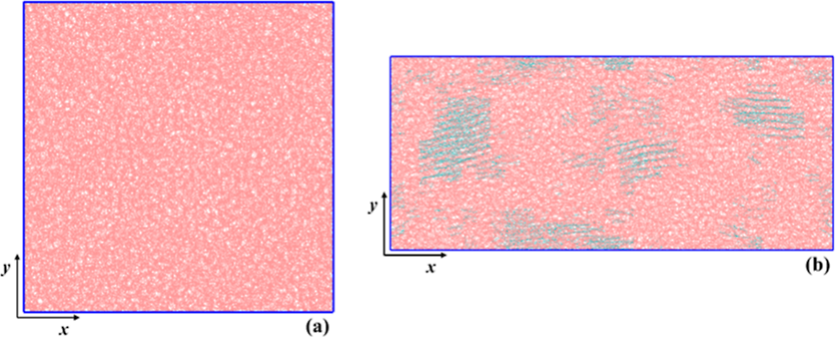
(a) Projections of initial undeformed purely amorphous PE configuration and (b) the same deformed state of the (semicrystalline) film at λ = 1.75. Amorphous and crystalline domains are denoted with pink and cyan colors, respectively. The snapshots were created using VMD software.^60,61^

We used the GROMACS simulation package to perform all of our quiescent and drawing MD simulations, applying a constant integration time step of Δ*t* = 2 fs. We also need to mention that, while the very high strain rate employed in our simulations does not correspond to real process strain rates, we are forced to adopt it due to the inevitable computational limitations of our united-atom simulations. Adopting such high strain rates is common practice in the recent simulation literature. The strain rates here have been carefully chosen so as to avoid unphysical phenomena, such as cavitation.^29^ In addition, there are complex effects of polymer dynamics taking place across different scales on the mechanism of crystallization, therefore on rates and morphology. We have estimated the entanglement relaxation time τ_e_, the Rouse time τ_R_, and the disengagement (longest relaxation) time τ_d_, for our linear PE melt at our simulation temperature, by extrapolating from extensive MD simulations of the same model we have performed in our group.^62^ This procedure is described in the Supporting Information. Here we have chosen to study two radically different cases, which we describe adopting the Deborah number De = τ*/t* = ε̇τ. Our two extremes correspond to quiescent conditions (De = 0) and to uniaxial elongational flow (De_d_ ≫ 1). In Table 1 we report the characteristic melt relaxation times for our simulations, and the corresponding De. The time scale of our flow simulations is commensurate with the Rouse time, governing chain stretching, and much shorter than the disengagement time, governing orientation and large-scale motion of the chains.

**Table 1:** Characteristic Melt Relaxation Times and Their Corresponding Deborah Numbers

*Τ* = 340 Κ, ε̇ = 10^7^ s^–1^	τ_e_ (ns)	τ_R_ (ns)	τ_d_ (ns)
relaxation time	5.45	500	21.230 × 10^3^
De	0.055	5.0	212.3

### 3.2 Estimation of the Degree of Crystallinity

The degree of crystallinity of a semicrystalline sample, *x*_c_, and its evolution are fundamental attributes of polymer crystallization. It can be computed using various definitions, including ones based on enthalpy, density, and segment orientation. We have developed a methodology for estimating *x*_c_ from the orientation of segments,^33^ based on the strategy proposed by Yamamoto.^30^ Specifically, we divide our simulation box into cubic cells of edge length *L*_cell_ = 2σ_max_ = 2 × 3.95 Å = 7.9 Å at each time instant. In each cell we consider all chord vectors having their midpoints in the cell, a chord being the vector connecting the midpoints of two adjacent skeletal bonds. We calculate the mean orientational order parameter of the cell as

1 $$P_{2}^{\left(Y\right)}=\frac{\left\langle 3\cos^{2}\theta_{i,j}-1\right\rangle }{2}$$

with θ_*i*,*j*_ being the angle between two chord vectors, averaging over all possible pairs of chord vectors in the cell. The degree of crystallinity *x*_c_ of the system is estimated as the percentage of cells with *P*_2_^(*Y*)^ > 0.7. This method for quantifying the degree of crystallinity has been compared against other methods, based on the enthalpy and the density of the semicrystalline system, yielding excellent agreement.^33^ The method also allows us to categorize the cells of the simulation box into amorphous and crystalline and label chain segments lying in these cells accordingly. Furthermore, we have developed a home-built cluster analysis algorithm, through which we can group adjacent crystalline cells into clusters (crystallites), identifying and tracking them throughout our MD crystallization simulations.

### 3.3 Shape and Orientation Analysis

Being able to determine and analyze the influence of the mode of crystallization, be it the absence or presence of a flow field, on the resulting semicrystalline morphology (average shape, size, and orientation of the crystallites and spatial correlations among them) is of paramount importance. The size and shape characteristics of unperturbed single chains have been studied meticulously using a random flight model by Šolc and Stockmayer.^63^ Earlier, Koyama had considered the effects of excluded volume interactions through an analytical approach.^64^ Yoon and Flory developed a theoretical approach to the distribution of chain segments in unperturbed chains of finite length, using in a realistic chain model.^65^ Theodorou and Suter^45^ extracted shape measures for unperturbed single chains of linear PP of various tacticities, representative of melts, through Monte Carlo sampling based on the rotational isomeric state model. The eigenvalues of the radius of gyration tensor can serve as shape measures for a polymer chain or, generally, for any body defined by a distribution of mass in three-dimensional space. A core target of the present work is to conduct a comprehensive study of the shape evolution of crystallites developing under quiescent and stretch-induced crystallization conditions in entangled PE melt systems. As mentioned in the previous subsection, our cluster analysis algorithm allows us to group neighboring crystalline cells into clusters (crystallites). These crystallites can be identified and tracked throughout our MD simulations.^33^ Here, having singled out the largest cluster in each one of our specimen configurations and knowing the positions of UAs constituting it, we can conveniently calculate, at each moment, its radius of gyration tensor **S**, with elements

2 $$S_{\alpha \beta }=\frac{1}{n}\sum_{i=1}^{n}\left(r_{i,\alpha }-r_{cm,\alpha }\right)\left(r_{i,\beta }-r_{cm,\beta }\right),\alpha ,\beta \in \left\{1,2,3\right\}$$

In eq 2, *n* is the total number of particles (methylene or methyl groups) constituting the cluster, **r**_*i*_ = (*r*_*i*,1_, *r*_*i*,2_,*r*_*i*,3_) is the position vector of particle *i* (1 ≤ *i* ≤ *n*) and **r**_cm_ = (*r*_cm,1_,*r*_cm,2_,*r*_cm,3_) is the position vector of the center of mass of the cluster

3 $$r_{cm}=\frac{1}{n}\sum_{i=1}^{n}r_{i}$$

Equations 2 and 3 are mathematically equivalent to the expressions

4 $$S_{\alpha \beta }=\frac{1}{2n^{2}}\sum_{i=1}^{n}\sum_{j=1}^{n}\left(r_{i,\alpha }-r_{j,\alpha }\right)\left(r_{i,\beta }-r_{j,\beta }\right)=\frac{1}{n^{2}}\sum_{i=1}^{n-1}\sum_{j=i+1}^{n}\left(r_{i,\alpha }-r_{j,\alpha }\right)\left(r_{i,\beta }-r_{j,\beta }\right)$$

We determine the principal axis system of the cluster by diagonalizing the radius of gyration tensor **S**, obtaining the three eigenvalues, Λ_1_^2^, Λ_2_^2^, Λ_3_^2^, in descending order. The corresponding eigenvectors, **v**_1_, **v**_2_, **v**_3_ define the principal axes of inertia of the cluster. This diagonalization maps the cluster to an ellipsoidal shape, with the square roots of the eigenvalues being proportional to the lengths of the corresponding ellipsoid semiaxes. The eigenvalues can be combined to calculate various quantities that describe how the particles constituting the body under study, in our case the UAs constituting the cluster, are distributed in space. The first invariant of **S** corresponds to the mean squared radius of gyration

5 $$R_{g}^{2}=Tr\left(S\right)=\Lambda_{1}^{2}+\Lambda_{2}^{2}+\Lambda_{3}^{2}$$

a measure of the average size of the specific cluster.

To further describe the ellipsoidal nature of our nuclei, we define asphericity as

6 $$\frac{b}{R_{g}^{2}}=\frac{\Lambda_{1}^{2}-\frac{1}{2}\left(\Lambda_{2}^{2}+\Lambda_{3}^{2}\right)}{\Lambda_{1}^{2}+\Lambda_{2}^{2}+\Lambda_{3}^{2}}$$

and acylindricity as

7 $$\frac{c}{R_{g}^{2}}=\frac{\Lambda_{2}^{2}-\Lambda_{3}^{2}}{\Lambda_{1}^{2}+\Lambda_{2}^{2}+\Lambda_{3}^{2}}$$

quantities useful in characterizing shape, both reduced by the squared radius of gyration. *b* = *c* = 0 corresponds to a particle distribution of tetrahedral or higher symmetry, while *c* = 0 indicates a particle distribution of cylindrical symmetry.

The orientation of the emerging crystalline phase can be quantified by employing the **Q**-tensor, which is widely used to describe nematic liquid crystals.^46,66^ As is the case with the radius of gyration tensor, the **Q**-tensor is also implemented locally, on the largest cluster. A definition of the **Q**-tensor can be given as

8 $$Q=\left\langle uu\right\rangle -\frac{I}{3}$$

where **u** is a unit chord vector, as defined in Section 3.2, **uu** is the dyadic formed from vector **u** and the average is taken over all chord vectors in the cluster. **I** is the unit tensor; with the definition above, **Q** is symmetric and traceless.

The diagonalization of the **Q**-tensor results in three eigenvalues, which must sum to zero. The largest eigenvalue, necessarily positive, is indicative of the degree of orientational correlation in the set of considered vectors **u**. It is usually symbolized as $\frac{2}{3}S_{Q}$ , with *S*_**Q**_ being a scalar order parameter.^46^ The unit eigenvector corresponding to the largest eigenvalue of **Q** is the director **n**_chords_, defining the direction of prevalent orientation of the vectors **u**. One can readily show that

9 $$S_{Q}=\frac{3}{2}\left\langle {\left(u\cdot n_{chords}\right)}^{2}\right\rangle -\frac{1}{2}$$

where **u**·**n**_chords_ is the cosine of the angle between one of the unit vectors and the director and the average is taken over all vectors in the set.

If the set of vectors **u** is characterized by perfect cylindrical symmetry around the director, the **Q**-tensor assumes the form $Q=diag\left(\frac{2}{3}S_{Q},-\frac{1}{3}S_{Q},-\frac{1}{3}S_{Q}\right)$ . Equation 9 remains valid, however, even in the absence of such perfect symmetry. Equation 9 can be considered a special case of the definition of a second-rank orientational order parameter (averaged second Legendre polynomial of the cosine) for a set of vectors with respect to a reference axis α

10 $$\left\langle P_{2}\left(\cos\theta^{\alpha }\right)\right\rangle =\frac{3}{2}\left\langle \cos^{2}\theta^{\alpha }\right\rangle -\frac{1}{2}$$

where θ^α^ is the angle between a vector in that set and the reference axis. For ⟨*P*_2_(cosθ^α^)⟩ = 1, the alignment between the vectors in the set and the reference axis is perfect; for ⟨*P*_2_(cosθ^α^)⟩ = 0, the alignment is absent, i.e. the vectors are randomly oriented with respect to the reference axis; finally, for ⟨*P*_2_(cosθ^α^)⟩ = −1/2, the prevalent alignment is perpendicular to the reference axis.

Equation 10 will be employed in several instances in this study. In the particular case of *S*_**Q**_ in eq 9, the reference axis is the local director **n**_chords_. Note that the *P*_2_ defined in eq 10 is different from the measure *P*_2_^(*Y*)^ that was introduced in the Yamamoto criterion, eq 1.

## 4 Results and Discussion

### 4.1 Degree of Crystallinity

In Figure 3a,b we display the evolution of the degree of crystallinity, *x*_c_, versus time for quiescent and stretch-induced PE crystallization, respectively. We can distinguish two separate regions in the plots. In the initial region, before nucleation, the system is predominantly amorphous. The degree of crystallinity is very close to zero and, while several crystal nuclei are formed, they quickly dissolve. The second region starts at the moment *x*_c_ begins to rise. We consider this point in time to be the induction time, indicating that nucleation is successful and crystal growth begins.

**Figure 3:**
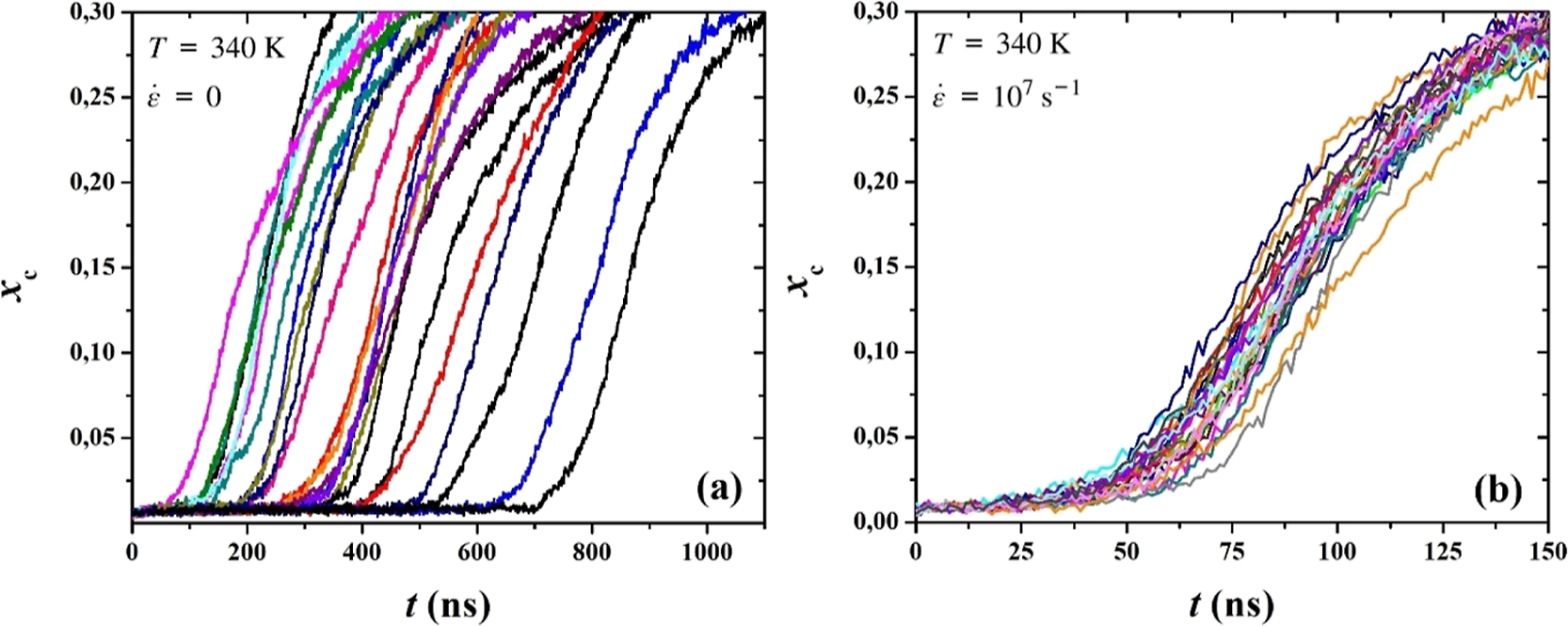
Degree of crystallinity, *x*_c_, as a function of time, for all PE systems, under (a) quiescent and (b) stretching conditions.

Two things are immediately noticeable: First, under the presence of an external flow field, crystallization becomes rapidly faster, as less than 50 ns are required for the samples to crystallize, which is shorter than the minimum time observed under quiescent conditions. Second, the wide scatter of induction times which is present under quiescent conditions is dramatically reduced under stretch-induced crystallization.

The above observations generate a multitude of questions on how kinetics and structure are affected by the presence or absence of a flow field during crystallization. Insight will be provided by probing the induction times through the radius of gyration of the largest crystal nucleus, by performing a mean first-passage time fit, and finally by employing an analysis of the largest cluster’s radius of gyration tensor.

### 4.2 Estimation of Induction Time from the Radius of Gyration

An estimation of the individual induction times of each simulation can be provided through plotting the time evolution of the squared radius of gyration of the largest ordered cluster, for each separate trajectory. We have measured the squared radius of gyration for the largest ordered cluster in our specimens, which we choose as the reaction coordinate throughout this study, using eq 2 or 4 along with eq 5. In Figure 4 we show the evolution of the root square radius of gyration, *R*_g_, and of the degree of crystallinity, *x*_*c*_, against time, estimated for a single PE system, under (a) quiescent and (b) stretching conditions. Three regions are determined: (i) an initial region, corresponding to the incubation period during which small nuclei form and dissolve; here *R*_g_ of the largest cluster fluctuates heavily around an approximately constant value; (ii) a second region, where the fluctuations instantly decrease and *R*_g_ starts to increase steadily, indicating the existence of a nucleus which has exceeded critical size and begins to grow; and (iii) a final region, during which a plateau is reached under quiescent conditions (a), while, on the other hand, the cluster’s *R*_g_ exhibits large fluctuations, jumps to a higher value and continues to grow under stretching conditions (b). In all cases, the point where the first region ends and the second one begins corresponds to the induction time, τ_i_*, for the particular trajectory *i*. This can readily be extracted from the *R*_g_(*t*) trace of every single trajectory analyzed, with reasonable certainty. Juxtaposition of the degree of crystallinity curve, which is calculated for the entire system, against the radius of gyration of the largest nucleus estimate provides us with a good qualitative agreement on the estimation of τ_i_*, verifying that nucleation in the bulk can be accurately described by adopting the size of the largest nucleus as a reaction coordinate. By visual observation of trajectories, we have established that the fluctuations observed after growth has started, evident in region (iii) of Figure 4b, can be attributed to the merging and splitting of crystalline clusters; the monitored largest cluster attempts, and in this particular example ultimately manages, to absorb other, smaller nearby clusters present in the system. The above-mentioned phenomenon is evident in the videos provided in the Supporting Information section. There we show a characteristic sequence of 3D representations, during which merging and splitting of crystalline clusters occurs under stretching.

**Figure 4:**
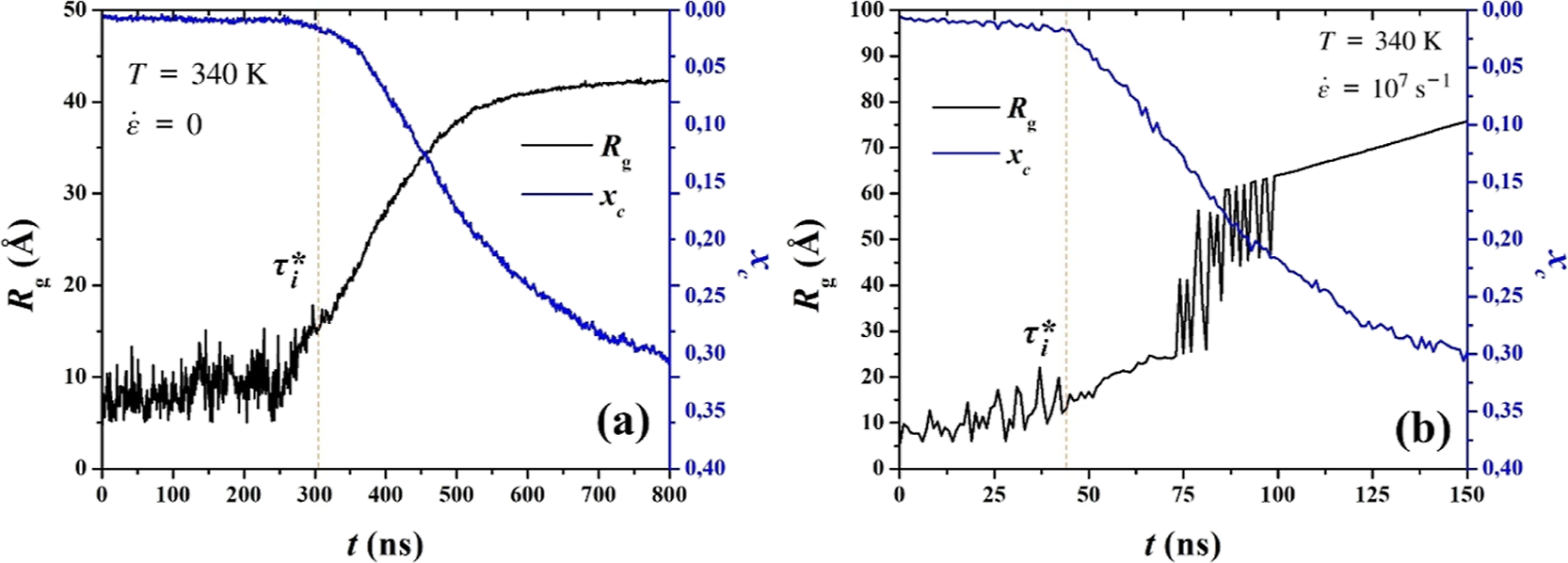
Radius of gyration of the largest ordered cluster, *R*_g_, as a function of time, juxtaposed with the degree of crystallinity, *x*_c_, calculated from a single trajectory under (a) quiescent and (b) stretching conditions. The dotted line intersects the plot at the corresponding trajectory’s induction time, τ_i_*.

### 4.3 Probability Distribution of Induction Times

In the previous section we described how we can estimate the induction time separately for each trajectory via the time evolution of the radius of gyration of the largest ordered cluster. Having applied this methodology to all our specimens, we have calculated the corresponding induction times, τ_i_*, for every trajectory. Nucleation under both quiescent and stretching conditions can be modeled by employing a log-normal distribution.^67,68^ This is due to nucleation appearing as a range of events with slightly different rate constants and activation free energies. By the law of large numbers, activation free energies would be normally distributed, leading to a log–normal distribution of rates. The cumulative log-normal distribution is given by

11 $$P\left(t\right)=\frac{1}{2}\left\{1+\mathrm{erf}\left[\frac{\ln\left(t\right)-\mu }{\sigma \sqrt{2}}\right]\right\}$$

μ and σ being parameters of the distribution, the expected value and standard deviation of the variable’s natural logarithm, respectively. In Figure 5 we showcase the fits of the cumulative log-normal distribution, to our data, under (a) quiescent and (b) stretch-induced crystallization. The fit quality is excellent in both cases. The average induction time can be calculated by the mean of the distribution

12 $$\tau_{dist}^{*}=\exp\left(\mu +\frac{\sigma^{2}}{2}\right)$$

**Figure 5:**
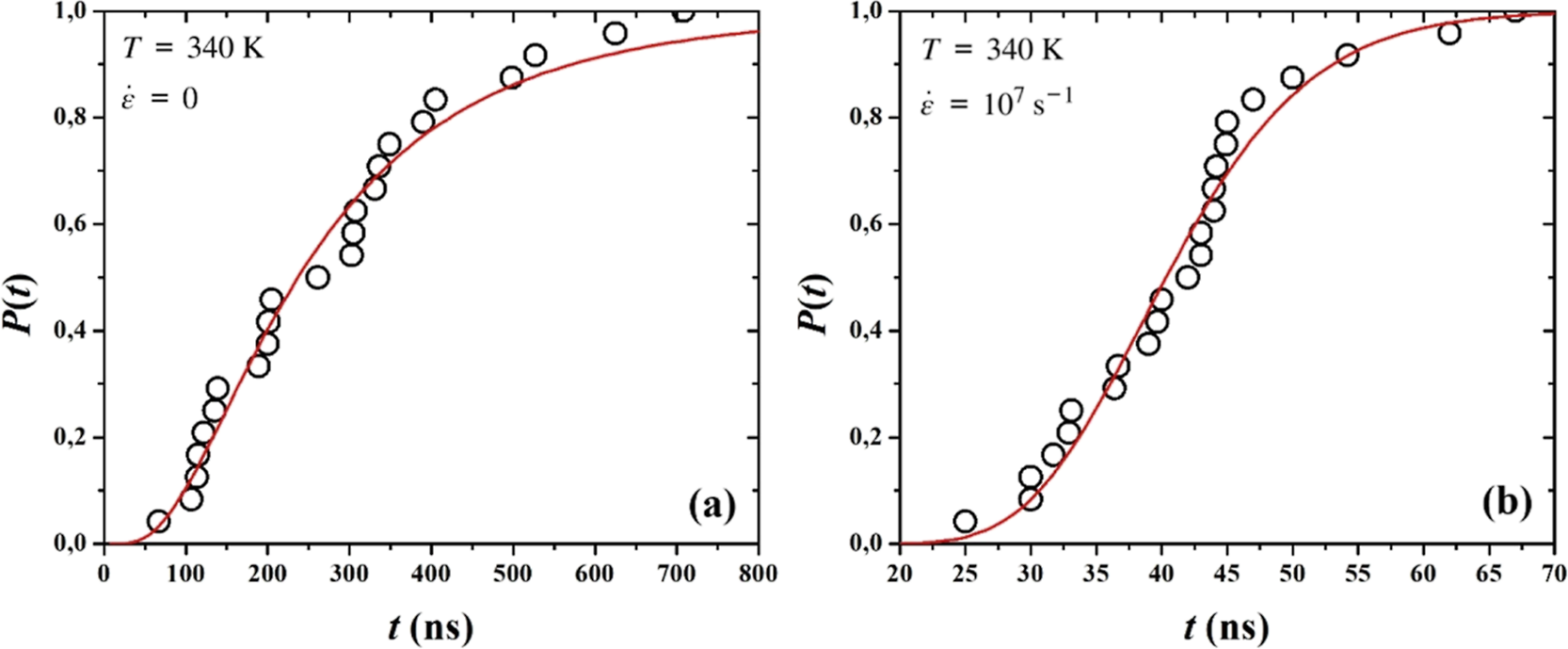
Cumulative log-normal probability distribution of the induction times under (a) quiescent and (b) stretch-induced crystallization.

As expected, it is significantly diminished upon stretching. The induction times calculated by the log-normal probability distribution fit are presented in Table 2, next to corresponding independently calculated induction times τ_avg_^*^, acquired through averaging the induction times τ_i_* over all individual trajectories.

**Table 2:** Average Induction Times Calculated From the Log-Normal Distribution and from Averaging over the 24 Different Individual Induction Times Calculated

$\mathrm{\varepsilon ̇}\;\left(s^{-1}\right)$	σ	τ_dist_^*^ (ns)	τ_avg_^*^ (ns)
0	0.69 ± 0.03	300.10 ± 1.02	289.19 ± 34.90
10^7^	0.21 ± 0.01	41.26 ± 1.00	41.87 ± 2.00

### 4.4 Mean First-Passage Time Analysis

In this section we perform a kinetic analysis of nucleation in our quiescent and stretch-induced crystallization simulations. We choose the size *n*_max_, in methylene units, of the largest ordered cluster present as our order parameter. The basic idea is that *n*_max_ executes overdamped Brownian motion on the free energy profile, possessing a barrier, that renders nucleation an infrequent event. Based on this idea we accumulate the mean, τ, over all trajectories, of the time that must elapse for *n*_max_ to reach a specific value for the first time. Considering different values of *n*_max_, we accumulate the functional dependence τ­(*n*_max_). Under certain conditions^21,42,69^ this dependence can be described by the equation

$$\tau \left(n_{\max}\right)=\frac{\tau_{MFP}^{*}}{2}\left[1+\mathrm{erf}\left(Z\sqrt{\pi }\left(n_{\max}-n^{*}\right)\right)\right]+\frac{G^{-1}}{2}\left(n_{\max}-n^{*})[1+\mathrm{erf}\left(C\left(n_{\max}-n^{*}\right)\right)\right]$$ 13

By fitting this equation to our simulation results we estimate key parameters describing crystal nucleation and growth. This is what is referred to as mean first-passage time (MFPT) analysis^25,42,54^ in the simulation literature.

In eq 13, τ_MFP_^*^ is the induction time, i.e., the time that needs to elapse before a critical nucleation event occurs, n* is the critical nucleus size, *Z* is the Zeldovich factor, which corresponds to the probability for a critical nucleus to survive and not dissolve back into the melt phase, and *G* is the growth rate (beyond nucleation). *C* is as a large positive number involved in approximating a Heaviside function through a continuous error function. It is set here to *C* = 1000, a value at which the rest of the fitting parameters are invariant with it.^25^ Finally, the induction time τ_MFP_^*^ is connected with the nucleation rate *I* via the equation

14 $$I=\frac{1}{\tau_{MFP}^{*}V}$$

where *V* is the volume of the system in the melt phase.

The fits of eq 13 to our data are shown in Figure 6, (a) for the quiescent and (b) for the stretch-induced crystallization, and the values obtained from the MFPT fits are displayed in Table 3. As expected, the induction time decreases significantly in the presence of a flow field, during stretch-induced crystallization, resulting in increased nucleation rate. Also, the induction times calculated by the MFPT fits are in very good agreement with the corresponding calculations by the log-normal probability distribution, presented in Table 2. The critical nucleus size is approximately the same under both conditions. The growth rate is also enhanced under flow. In stretch-induced crystallization we observe a change in the slope, postnucleation, identifying two separate growth regions, something not happening during quiescent crystallization. This can be attributed to the merging of clusters, as discussed above. During quiescent crystallization, under the observed time frame for the MFPT analysis, there is usually a single large nucleus, while under stretching, several clusters emerge, as shown in Figures 7 and 8, respectively. Some time after nucleation, these clusters begin to merge with each other, resulting in this dual slope regime during growth, with the change in slope observed at *t* ≈ 70 ns. To determine the change in growth rate, we perform an additional linear fit in this second region, the inverted slope being the accelerated growth rate, *G*_2_, determined at 347.2 ± 20 UAs/ns. These phenomena will be investigated thoroughly in the rest of our analysis.

**Figure 6:**
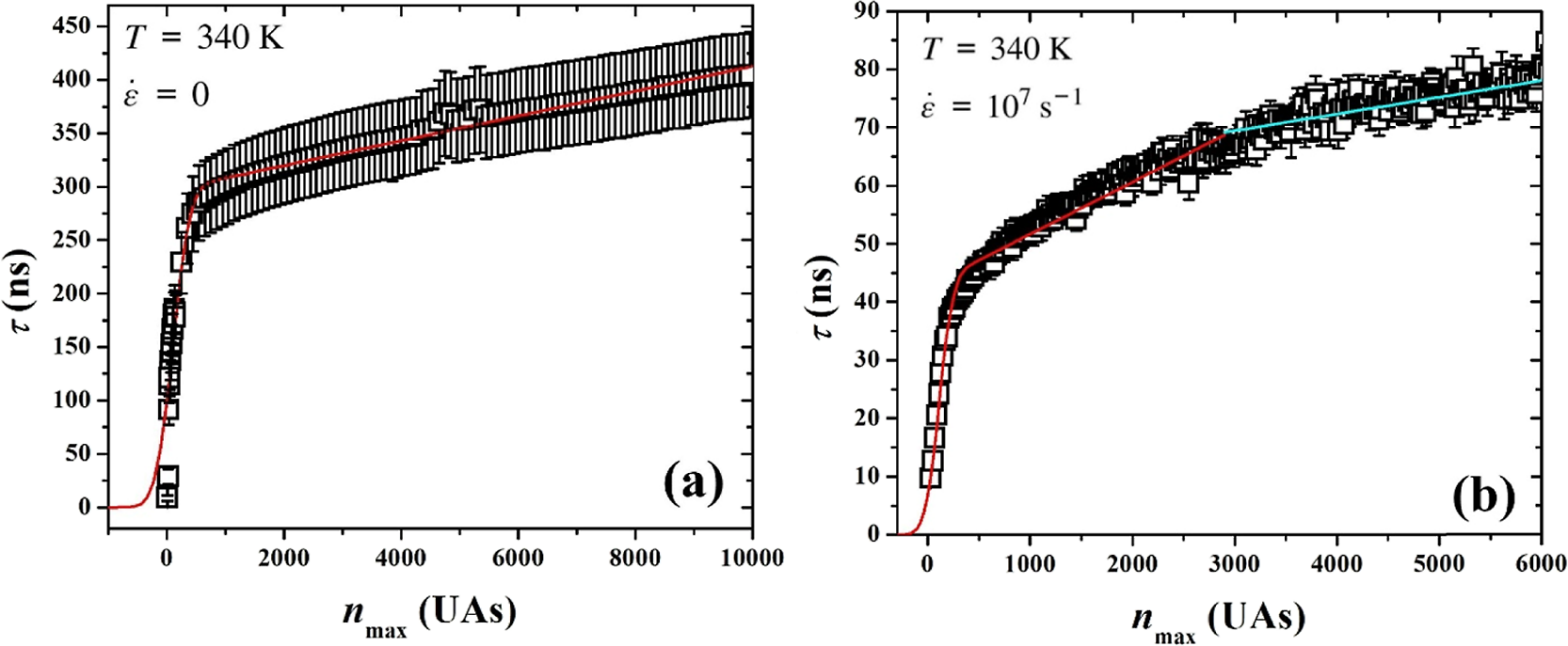
Mean first-passage time, averaged over all 24 simulation runs in each case, with the corresponding error bars, as a function of the size of the largest nucleus expressed in number of united atoms, and the corresponding MFPT theoretical fit (red line), under (a) quiescent and (b) stretching conditions, including a second, linear fit (cyan line), for the change of slope along the growth region under stretching.

**Table 3:** Mean First-Passage Time Analysis Parameter Values

$\mathrm{\varepsilon ̇}\;\left(s^{-1}\right)$	*Z* × 10^3^	n* (UAs)	τ_MFP_^*^ (ns)	*I* (10^25^ cm^–3^ s^–1^)	*G* (UAs/ns)
0	1.74 ± 0.07	100 ± 4	298 ± 1	0.12 ± 0.00	85.6 ± 1.5
10^7^	3.68 ± 0.14	106 ± 3	44 ± 0	0.82 ± 0.00	111.5 ± 2.5

**Figure 7:**
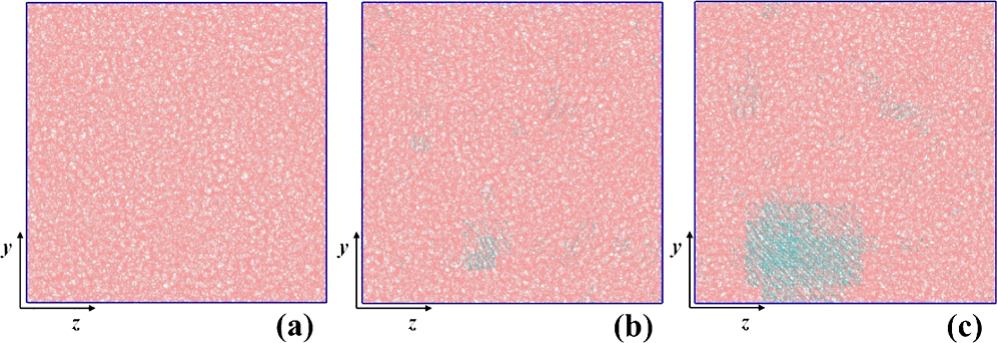
Snapshots of a semicrystalline PE system generated under quiescent conditions, at three different times, (a) *t*_0_ = 0, (b) *t*_1_ = τ_i_* = 475 ns and (c) *t*_2_ = 600 ns. Amorphous and crystalline chain segments are denoted with pink and cyan colors, respectively. The temperature is 340 K. The snapshots were created using VMD software.^60,61^

**Figure 8:**
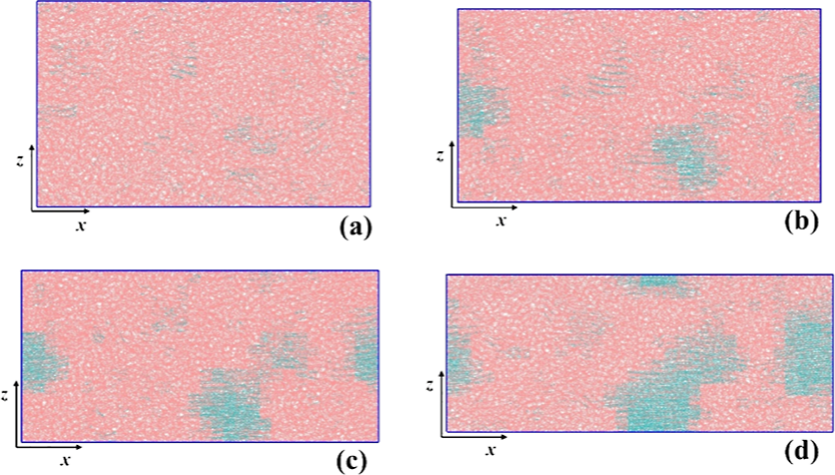
Snapshots of a semicrystalline PE system generated under stretching, at four different time instants, (a) *t*_1_ = τ_i_* = 40 ns, (b) *t*_2_ = 55 ns, (c) *t*_3_ = 65 ns and (d) *t*_4_ = 85 ns. Amorphous and crystalline chain segments are denoted with pink and cyan colors, respectively. The temperature is 340 K. The snapshots were created using VMD software.^60,61^

In Figures 7 and 8 we showcase snapshots of two indicative PE semicrystalline specimens, one created under quiescent conditions and the other one under stretching, respectively. Each snapshot corresponds to specific times along each corresponding trajectory. One can easily note that in the quiescent case, Figure 7, there is no specific orientation of the crystalline regions and also that the majority of the crystalline phase concentrates in a single nucleus. In Figure 8, on the other hand, the clear orientation of the nascent crystalline phase along the stretching direction is evident, while a very notable emergence of multiple nuclei occurs. Furthermore, in Figure 8c,d, one can discern a “supercluster” formed, resulting in the accelerated growth rate observed under stretching.

It must be mentioned that chain configuration is heavily influenced by stretching, catalyzing nucleation and growth. The presence of a deformation field results in elongated chains, with segments heavily oriented along the stretching axis. The chains uncoil rapidly as the process progresses. On the other hand, under quiescent crystallization the chains remain coiled and there is minimal change in their radius of gyration. Chain folding is observed in both cases. The time evolution of the averaged chain radius of gyration is featured in Figure S2. Illuminating videos of a single PE chain undergoing quiescent and stretch-induced crystallization are also featured in the Supporting Information section (Sections S10 and S11).

### 4.5 Crystal Nucleus Shape Analysis

As mentioned in the Methodology section, by forming and diagonalizing the radius of gyration tensor of the crystallites, we are able to determine and track their shape evolution. In this study, we focus only on the largest ordered cluster present in each one of our specimens and compare between the quiescent and stretch-induced crystallization scenarios. The mean squared radius of gyration, asphericity and acylindricity are all calculated averaging over all 24 trajectories of each case. To make averaging possible for our quiescent simulations, we shift the independent variable, time, by τ_i_*, for each trajectory involved. This results in negative times, representing the incubation period, and positive times, where nucleation has occurred and crystal growth is taking place.

#### 4.5.1 Mean Squared Radius of Gyration

Our analysis begins with the time evolution of the mean squared radius of gyration of the largest crystallite, shown in Figure 9. The effects of flow on the size of crystallites are immediately distinct. The incubation period is much shorter under the stretch-induced crystallization and postnucleation ${\left\langle R_{g}^{2}\right\rangle }^{1/2}$ shows a much faster increase compared to the quiescent case. The terminal value of ${\left\langle R_{g}^{2}\right\rangle }^{1/2}$ is larger under drawing conditions. The plateau value at long times corresponds to a percolated network of clusters that has ceased growing.

**Figure 9:**
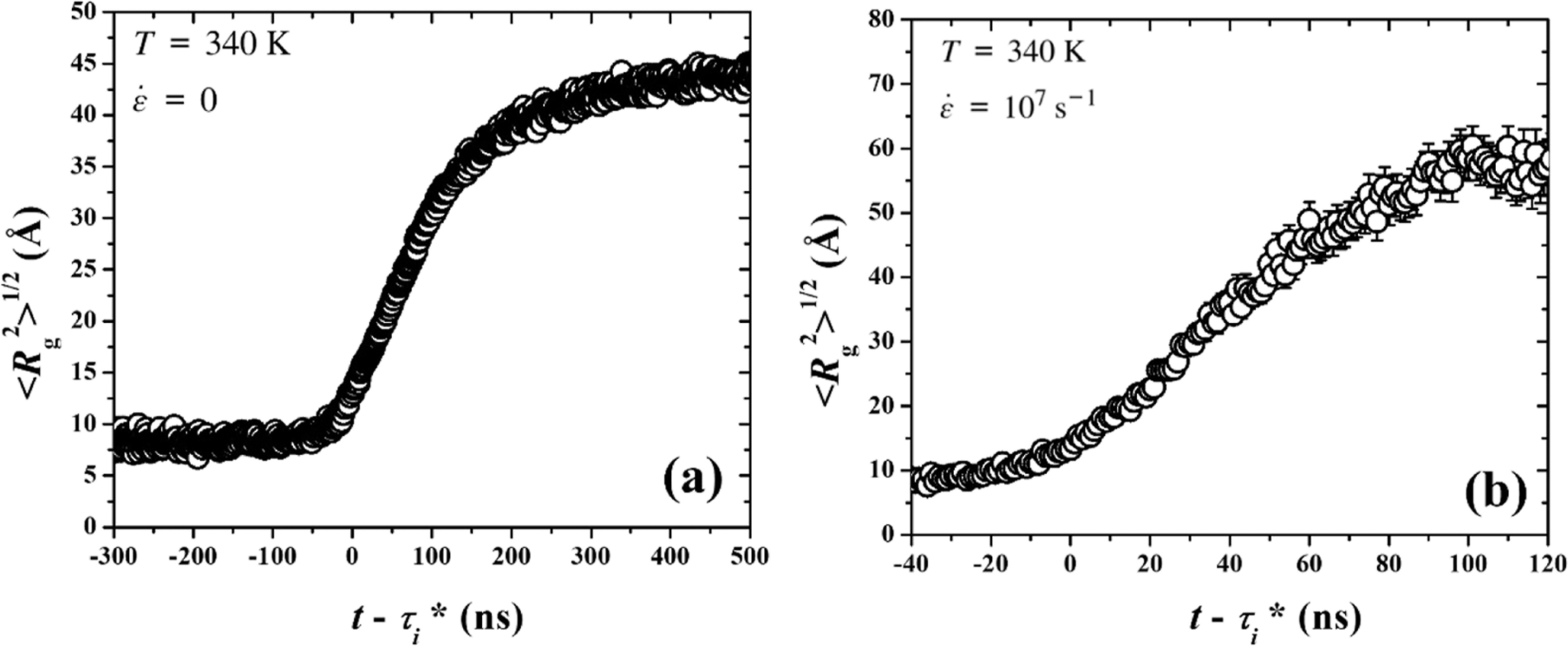
Root mean square radius of gyration, averaged over the total of our simulations, as a function of time shifted by τ*, under (a) quiescent and (b) stretching conditions.

#### 4.5.2 Asphericity, Acylindricity and Orientation

Moving on, in Figure 10 we present the time evolution of both reduced asphericity, ⟨*b*/*R*_g_^2^⟩, and reduced acylindricity, ⟨*c*/*R*_g_^2^⟩, averaged over the total of our (a) quiescent and (b) stretching simulations. Here, a very noticeable difference is seen between quiescent and drawing simulations. In the quiescent case, the crystallites initially show high asphericity and low acylindricity during nucleation, with relatively large fluctuations reflecting the instability of the forming nucleus. At the time of nucleation, however, the fluctuations become much more limited, acylindricity slightly decreases, arriving at a lower terminal value, while asphericity decreases sharply and, several hundred ns later, reaches a plateau, signifying that the crystallite shape is stabilized, adopting a predominantly spherical symmetry. Under stretching, while acylindricity initially follows a relatively similar pattern to the quiescent case, reaching a clearer plateau at an even lower terminal value, asphericity exhibits a totally different behavior. Starting from a maximum value, it decreases as nucleation occurs, but then, after displaying a minimum at 30 ns after nucleation, it increases again to adopt a terminal value of 0.6. The minimum coincides with the change in growth rate pointed out in our MFPT analysis. The above signify that a completely different crystallite geometry is formed under strain, of predominantly cylindrical shape.

**Figure 10:**
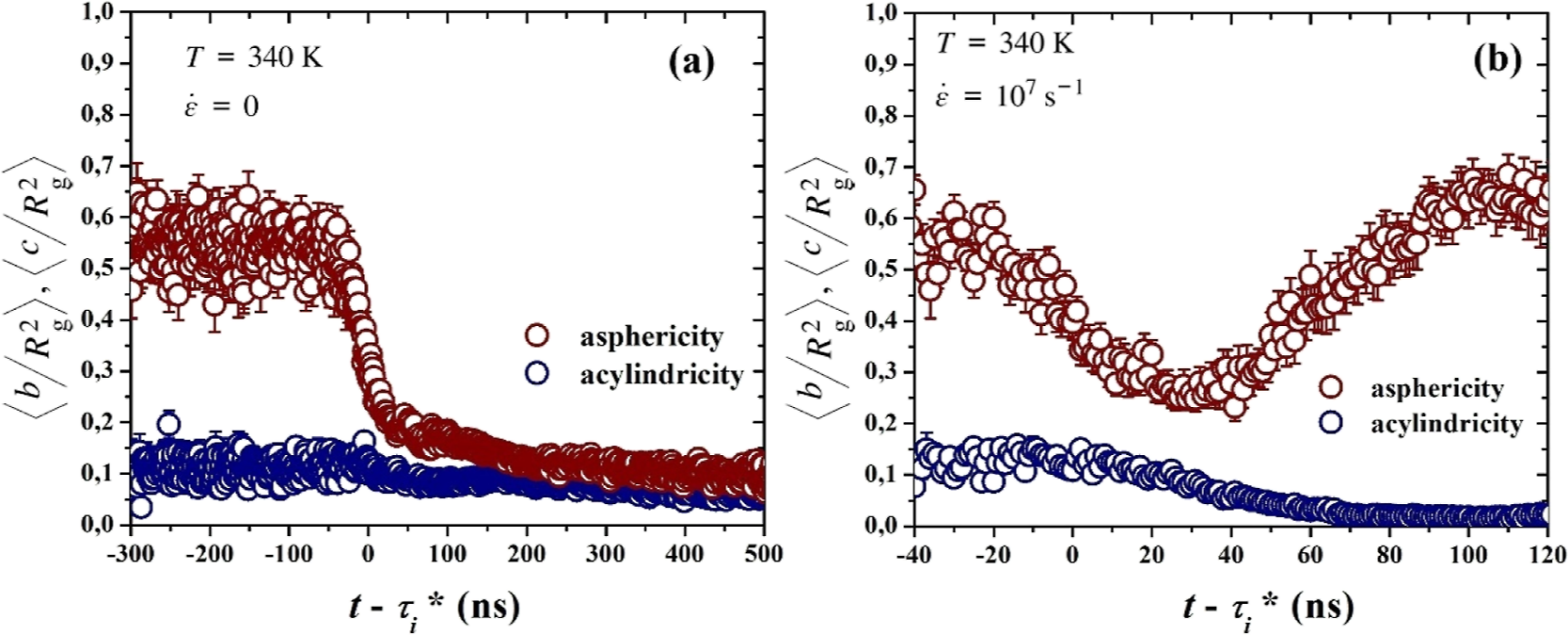
Mean reduced asphericity and reduced acylindricity, presented as functions of time shifted by τ*, averaged over all of our simulations, under (a) quiescent and (b) stretching conditions, respectively.

Having offered insight into the shape of the emerging clusters, we now proceed to shed light on the orientation of those crystalline domains, regarding both overall cluster orientation and chord orientation. By calculating the dot products of the primary eigenvector of the diagonalized radius of gyration tensor with each of the unit vectors along the three axes of the coordinate frame, and adopting a second Legendre polynomial formalism (see eq 10), we can quantify the distinction between how cluster orientation occurs under quiescent, versus under stretching conditions. In Figure 11 we present our findings for (a) quiescent and (b) stretch-induced crystallization. In the first case, *P*_2_ acquires values fluctuating around zero, showing no preference of orientation, during both nucleation and growth. The slightly elevated values observed along the *y*-axis can be attributed to the random nature of orientation and this deviation is deemed acceptable, as 0 is within error bars. For an augmented sample of uncorrelated initial melt configurations, all three *P*_2_ would converge to 0. Under stretching conditions, the case is radically different. While initially random, the emerging cluster adopts a preferred orientation, that of the stretching axis. The time instant when this distinction occurs, coincides with the characteristic time pointed out in the change of growth rate and with the asphericity minimum.

**Figure 11:**
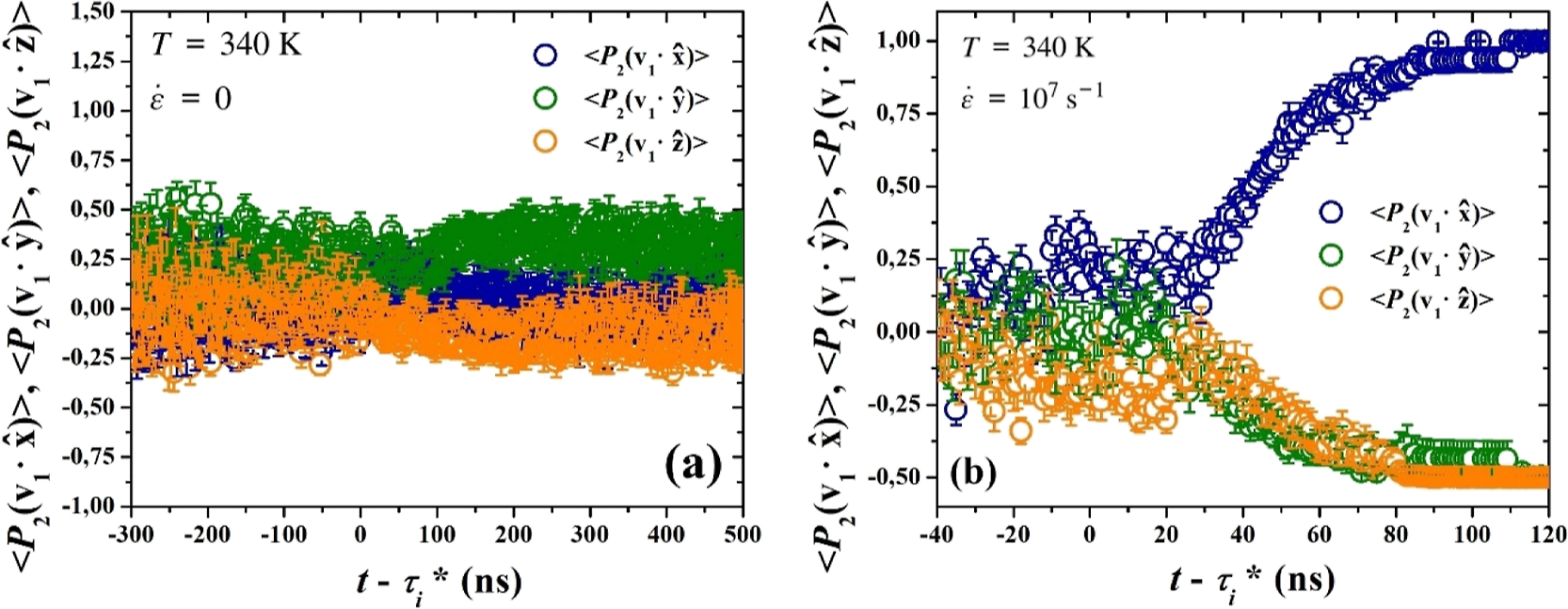
Second Legendre polynomials, *P*_2_, of the absolute dot product between the primary unit eigenvector of the largest cluster, **v**_1_, and the unit vectors along the three axes of the coordinate frame, presented as functions of time shifted by τ_i_* and averaged over all trajectories, under (a) quiescent and (b) stretching conditions, respectively.

In Figure 12 we present the scalar order parameter, *S*_**Q**_, for (a) quiescent and (b) stretch-induced crystallization. The difference between the two different simulation protocols is, again, striking. Under quiescent conditions, *S*_**Q**_ fluctuates around values close to 0, signifying that the chain orientation in the emerging crystalline phase is random and isotropic, throughout nucleation and growth. On the other hand, under stretching conditions, the effect of the deformation field is on full display from the early moments of nucleation, as the chords are rapidly oriented along the stretching axis, reaching a plateau region at *S*_**Q**_ = 0.94, showcasing a very highly oriented and anisotropic crystalline state.

**Figure 12:**
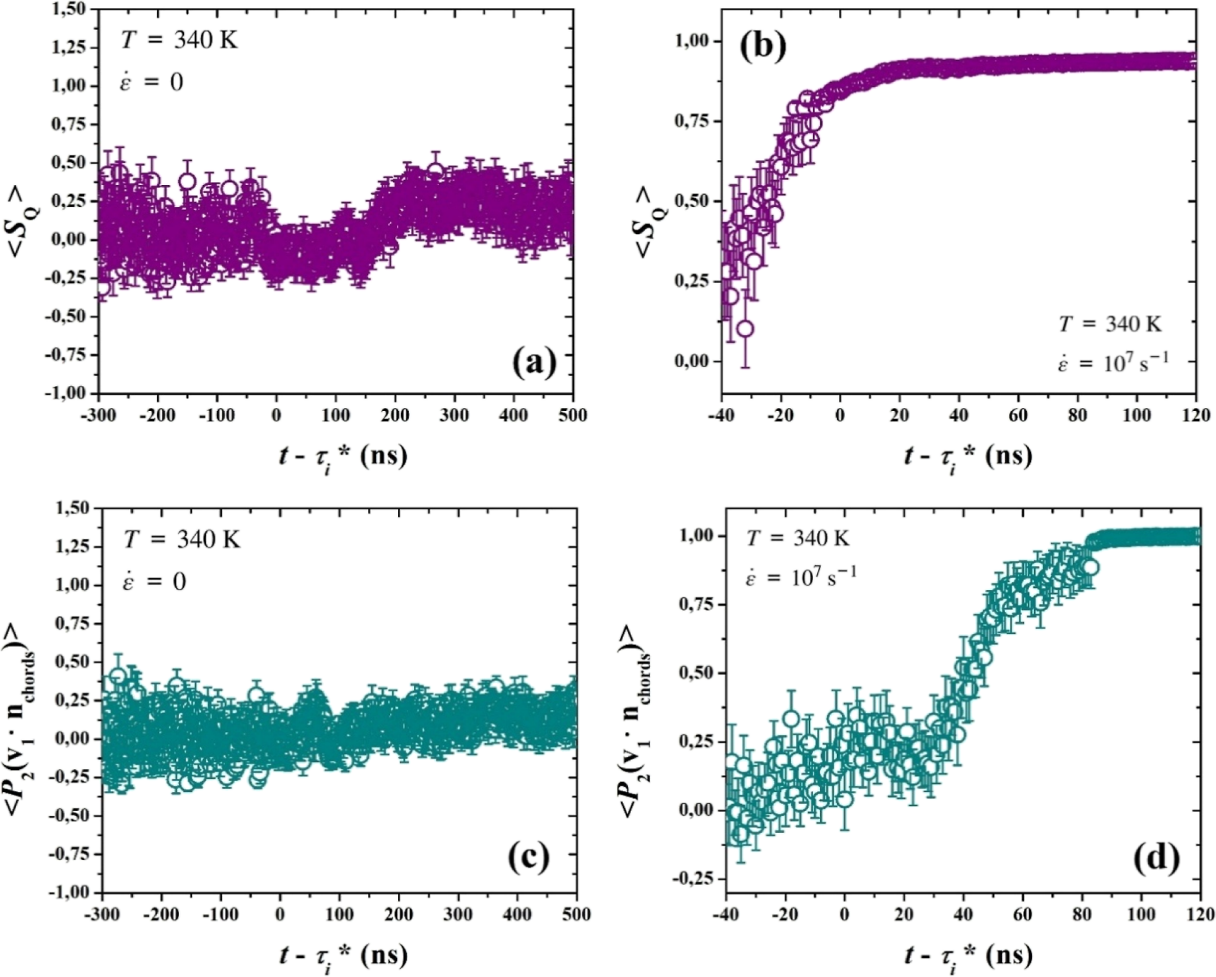
(top) Scalar order parameter, *S*_**Q**_, presented as a function of time shifted by τ* and averaged over all trajectories, under (a) quiescent and (b) stretching conditions, respectively. (bottom) Second legendre polynomials, *P*_2_, of the dot product between the primary unit eigenvector of the largest cluster, **v**_1_, and the chord director vector, **n**_chords_, in the same cluster, presented as functions of time shifted by τ_i_* and averaged over all trajectories, under (c) quiescent and (d) stretching conditions, respectively.

Finally, in the bottom part of Figure 12 we correlate the radius of gyration tensor primary unit eigenvector, **v**_1_, with the chord director vector **n**_chords_ acquired by the diagonalization of the local **Q**-tensor, combining both shape and chord orientation, under (a) quiescent and (b) stretching conditions. Again, we employ a second Legendre polynomial formalism, akin to eq 10. The two quantities are uncorrelated under quiescent conditions; no tendency is detected for the longest dimension of an ordered cluster to be parallel or perpendicular to the main direction of the chain stems constituting the cluster. On the other hand, for stretch-induced crystallization, although cluster shape and chain orientation are initially mostly uncorrelated, an increasing tendency for the cluster to be longer along the direction of orientation of the chains is observed up to and past nucleation. *P*_2_(**v**_1_·**n**_chords_) drops as the point of maximum sphericity of the crystallites is approached (compare Figure 10b). It then exhibits a very rapid increase starting at 30 ns post nucleation; as mentioned above, large cylindrical clusters are created through mergers of smaller clusters in this regime, and these large clusters are strongly oriented in the stretching direction. Finally, a plateau is reached, signifying a final structure where both the longest dimension of the cluster and the chord director align with each other and with the pulling direction (compare Figure 11b).

In Figures 13 and 14 we show snapshots of critical nuclei and their different shapes as crystallization progresses, for quiescent and stretch-induced crystallization, respectively, providing visualizations of the above calculated quantities of shape and orientation. From quiescent crystallization we display the largest nucleus (a) at a prenucleation stage, (b) at the induction time and (c) at a developed state. One can immediately note that the chord orientation at the prenucleation stage is not carried over to the fully grown nucleus and that the final orientation of the crystallite exhibits a chain tilt between the chain stems, which can be identified by **n**_chords_ in our work, and the normal to the lamellar plane, which we can identify as **v**_1_. Our nuclei adopt a predominantly spherical shape by the end of our quiescent simulations. Although no merging can be systematically identified between clusters in our quiescent simulations, we cannot state that coalescence of adjacent nuclei does not take place under quiescent conditions. This phenomenon has been observed in past simulation works,^70,71^ for end-confined^70^ and local structure order assisted systems.^71^ In our quiescent simulations, it is particularly hard for mergers to occur, as in the majority of our simulations the appearance of crystal nuclei is extremely sparse before a lamella will grow across the simulation box and will impinge upon periodic images of itself. An investigation on morphological features observed in long simulation times under quiescent conditions in some of our simulations will be presented in Section 4.7.

**Figure 13:**
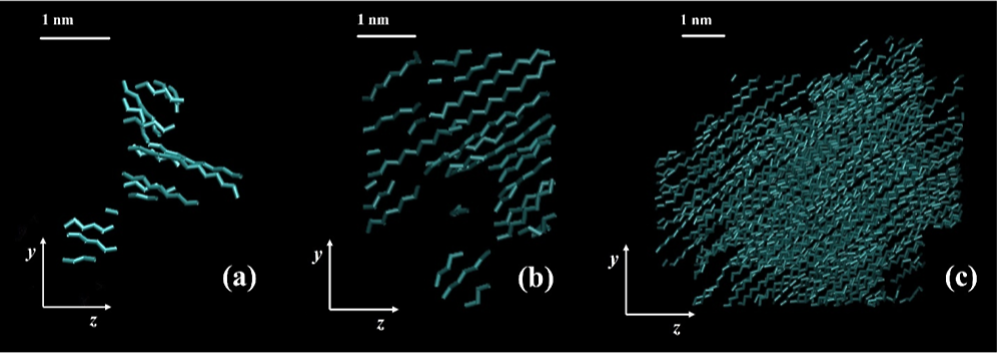
Snapshots of a largest crystalline nucleus generated under quiescent conditions, at three different times, (a) *t*_0_ = 300 ns, (b) *t*_1_ = τ_i_* = 475 ns and (c) *t*_2_ = 600 ns. The temperature is 340 K. The snapshots were created using VMD software.^60,61^

**Figure 14:**
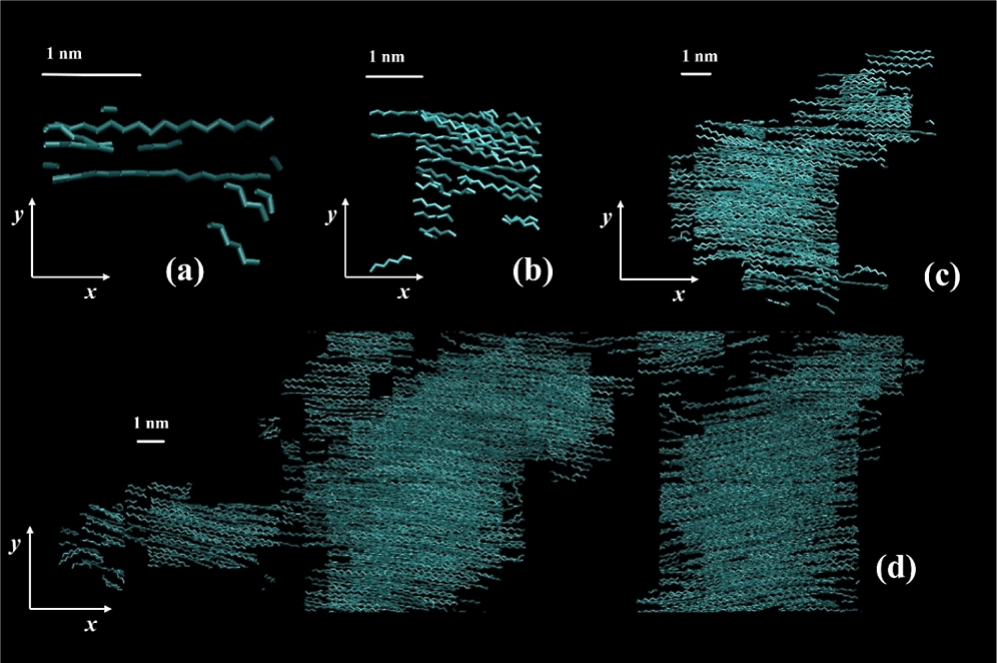
Snapshots of a largest crystalline nucleus generated under stretching, at four different times, (a) *t*_0_ = 20 ns, (b) *t*_1_ = τ_i_* = 41 ns, (c) *t*_2_ = 65 ns and (d) *t*_3_ = 120 ns. The temperature is 340 K. The snapshots were created using VMD software.^60,61^

Under stretching the situation is different. Again, we display the largest nucleus (a) at a prenucleation stage, (b) at the induction time, (c) at the asphericity minimum and (d) past that minimum. Even at the prenucleation stage, the effect of flow on the orientation of the crystalline regions is evident, as the chord vectors are heavily aligned with the stretching axis. As in quiescent crystallization, the nucleus adopts near spherical symmetry soon after nucleation, however the crystalline domains are highly oriented along the stretching axis, *x*. In Figure 14c we begin to see the deviation from spherical symmetry, as a second cluster attaches to the top right part of the nucleus. The final strongly cylindrical symmetry is showcased in Figure 14d, where nearby crystallites have merged with the largest nucleus, forming a supercluster, with all crystalline domains heavily oriented along the stretching direction, *x*. This merging behavior has been pointed out in a previous simulation study^47^ and has been proposed as a mechanism of shish crystal morphology formation.

### 4.6 Crystal Growth Analysis

By determining the eigenvalues of the mean radius of gyration tensor, we can define the shape evolution of the largest cluster, represented by an ellipsoid whose principal axes are proportional to the square roots of the eigenvalues, Λ_*i*_. In Figure 15 we display the evolution of the Λ_*i*_, showing the preferred shape evolution of the crystallite along its three principal directions. Again, we note the different regimes corresponding to nucleation and growth. Initially, there are fluctuations around a constant value, until a short time before nucleation where an increase is observed. The curves again demonstrate a sigmoidal shape, achieving a plateau at long times, except the one corresponding to the first principal direction under stretching. Under quiescent conditions there is no preferred orientation, as the crystalline regions are generated with random orientation and go on to grow more or less isotropically, resulting in a quasi-spherical cluster, with almost equal semiaxis lengths. In stretch-induced crystallization, however, although all three dimensions initially display similar behavior with the quiescent case, at about 30 ns after nucleation, the dimension along the first principal direction becomes highly favored, continuing to grow, while the other two directions remain practically constant. Notably, this time instant coincides with the minimum detected in the asphericity plot and the change of slope (growth rate) in our MFPT analysis. The semiaxis length observed is proportional to the lamellar thickness. It must be mentioned that the anisotropy of nucleation and growth -with the exception of the cylindrical shape under stretch-induced crystallization- is not evident in this form of the Λ_*i*_ plots. We provide an illuminating figure in the Supporting Information section, Figure S4, where we demonstrate a treatment of the Λ_*i*_ which makes the anisotropy showcased in Figure 10, apparent. By looking at the relative difference between the rooted eigenvalues as indicators of length along the three principal directions of a cluster, we can confidently say that nucleation is not an isotropic phenomenon. Under both quiescent and stretching conditions, the nucleus starts off as an anisotropic stacking of cylindrical elements, exhibited in Figures 13 and 14. A significant decline in anisotropy is observed around nucleation, for both quiescent and stretch-induced conditions; the initially cylindrical clusters grow quickly in the direction normal to the stems, leading to a more spherical shape. In the quiescent case, the nucleus goes on to adopt the quasi-spherical symmetry observed in the 3D representations and quantified in Figure 10a. Under stretching, however, the tendency to adopt a more spherical symmetry is suddenly aborted, coinciding with the minimum in Figure 10b, because of the mergers between different crystalline clusters in the system. In addition to plotting the evolution of the square roots of the eigenvalues against time, we have also conducted linear fits, at the middle growth section. The slopes are proportional to the individual growth rates along the three principal directions. The linear fits and the values of the fitting parameters are presented in the Supporting Information, Section S3. As expected, the growth rate is isotropic for quiescent crystallization and slower than the rates observed under stretching. Growth under stretching is initially isotropic, but at *t* = 25 ns post nucleation growth becomes heavily favored along the stretching axis, *x*, over the *y* and *z* axis, a phenomenon attributed to the merging of clusters throughout this work.

**Figure 15:**
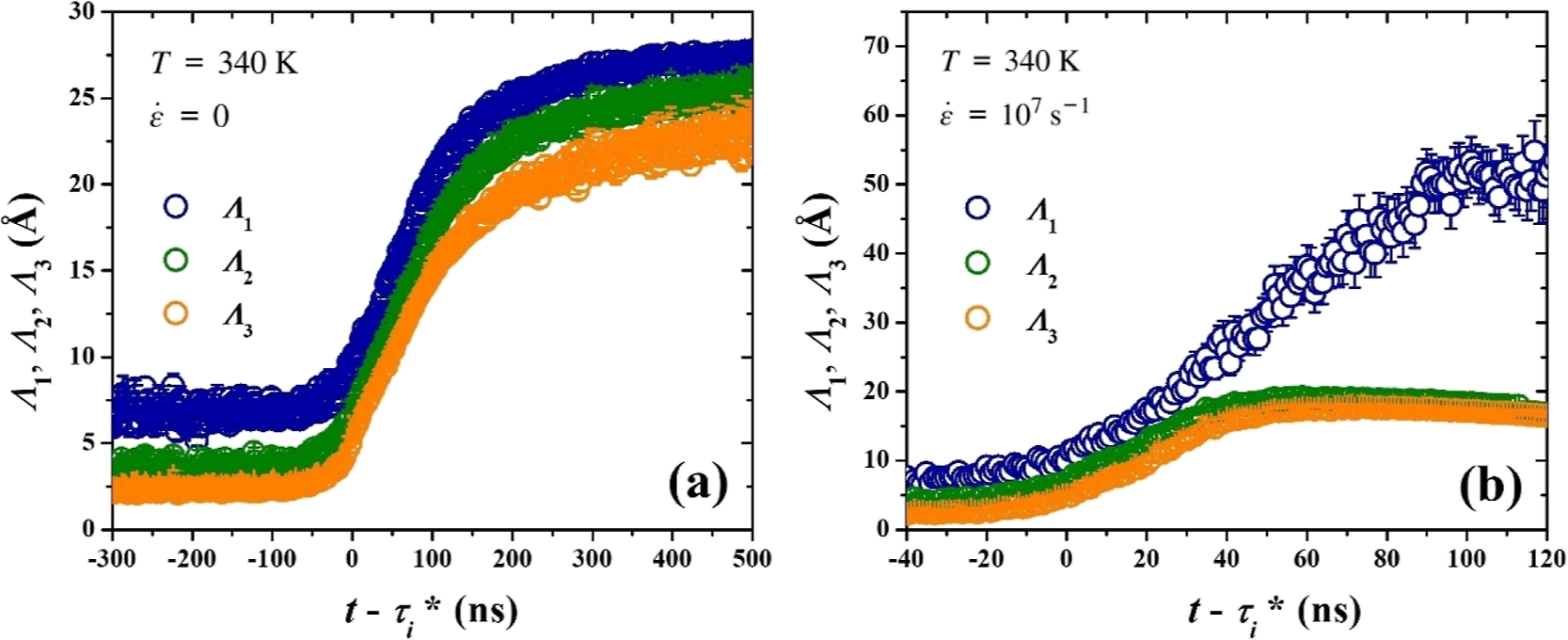
Square roots of the eigenvalues of the mean radius of gyration tensor, Λ_1_, Λ_2_ and Λ_3_, as functions of time shifted by τ_i_*, averaged over all trajectories, for (a) quiescent and (b) stretch-induced crystallization.

Based on these results, we can ascertain a volumetric growth rate, by first determining a proportionality constant. The volume of the ellipsoid representing the cluster is given by

15 $$V_{ellipsoid}=C_{vol}\frac{4}{3}\pi \Lambda_{1}\Lambda_{2}\Lambda_{3}$$

*C*_vol_ being a proportionality constant. By plotting the volume of the largest cluster, obtained through our cluster analysis algorithm as the sum of the volumes of all crystalline subcells constituting the cluster, against $\frac{4}{3}\pi \Lambda_{1}\Lambda_{2}\Lambda_{3}$ , we can conduct a linear fit, the slope of which is *C*_vol_. The fit is shown in Figure S5 of the Supporting Information. The proportionality constant was determined at *C*_vol,q_ = 10 for the quiescent case and at *C*_vol,s_ = 7, for the stretching case. The two different values are attributed to the different degrees of compactness exhibited by the largest cluster under the two scenarios. Under quiescent conditions, the final cluster is a compact crystalline cluster with a quasispherical symmetry (Figure 13c); under stretching, on the other hand, the final cluster results from mergers between lamellae, adopting a cylindrical symmetry overall, but with many hollow areas present (Figure 14d). In Figure 16 we display the volume of the largest cluster, using eq 15 for (a) the quiescent and (b) stretching cases. By performing a linear fit in the sections where the growth rate was calculated via the MFPT method, we define a slope, determined as a volumetric growth rate.

**Figure 16:**
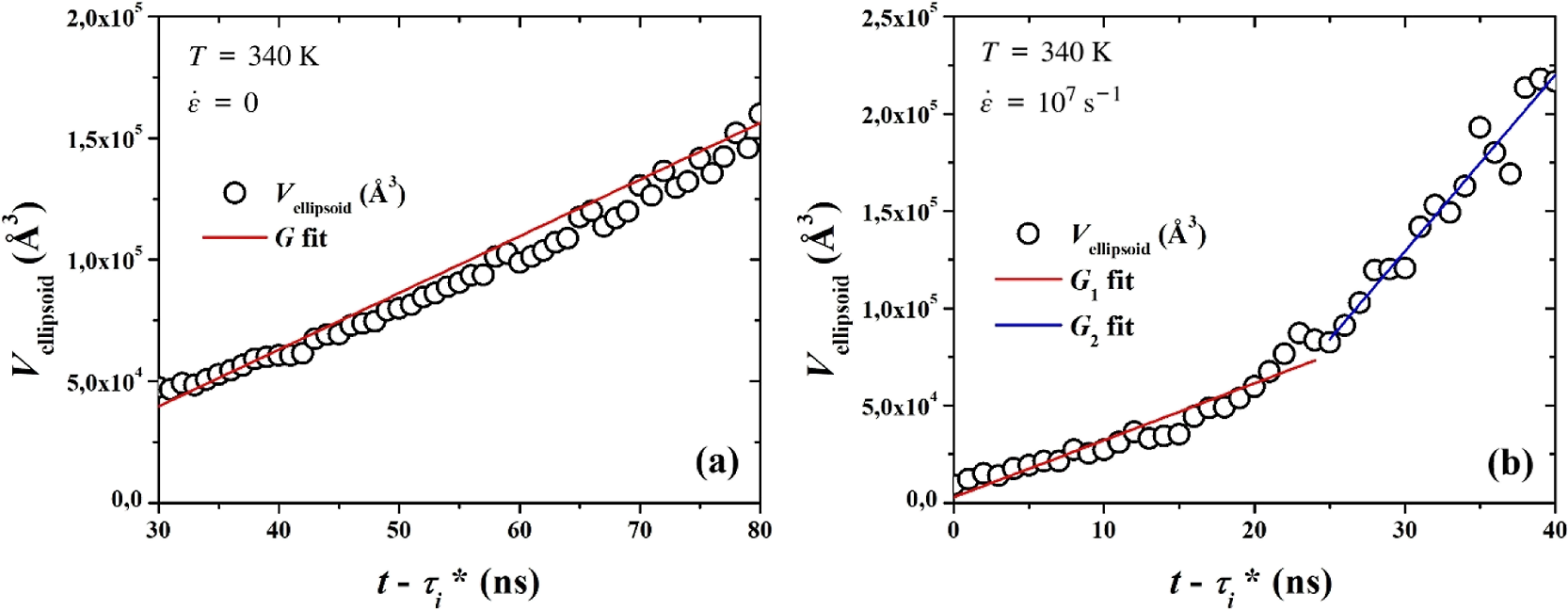
Ellipsoid’s volume, against time shifted by τ_i_*, and linear fits, the slope of which expresses a volumetric growth rate for (a) quiescent and (b) stretch-induced crystallization.

The growth rates determined here are then compared against the MFPT estimates, after unit conversion. The volume of a methylene unit in the nucleus is $\frac{M_{CH_{2}}}{N_{Avo}\rho_{n}}$ with $M_{CH_{2}}$ = 0.014 kg/mol, *N*_Avo_ being Avogadro’s number, and ρ_*n*_ being the mass density of the nucleus. For ρ_*n*_ = 850 kg/mol, the volume of a methylene unit $\frac{M_{CH_{2}}}{N_{Avo}\rho_{n}}$ is estimated as 27.35 × 10^–30^ m^3^ = 27.35 Å^3^. The volume of a cluster consisting of *n*_max_ methylenes is thus $\frac{M_{CH_{2}}}{N_{Avo}\rho_{n}}n_{\max}$ according to the MFPT analysis. The results, shown in Table 4, show very good coherence between MFPT and our geometric approach based on the radius of gyration tensor.

**Table 4:** Growth Rates, Estimated via the MFPT and the Ellipsoid’s Volume

system	*G*_MFPT_ (UAs/ns)	*G*_ellipsoid_ (UAs/ns)
quiescent	85.6 ± 1.5	85.2 ± 1.6
stretching—1st growth rate	111.5 ± 2.5	107.2 ± 7.0
stretching—2nd growth rate	347.2 ± 20	331.6 ± 19.6

A computational estimate for the growth rate, *G*, under quiescent conditions, at *T* = 340 K and using the same force field, can be extracted from the work of Verho et al.,^31^ yielding *G* ≈ 52 UAs/ns, in the same order of magnitude as our own estimates. However, it must be noted that crystallization in that work is initiated from seeds at 340 K, while our system is completely amorphous at the start of our simulations.

### 4.7 Structure and Morphology of Crystal Nuclei at Long Simulation Times under Quiescent Conditions

So far in this study we limited our focal point on time scales during which primary nucleation and growth occur, stopping the quantification of parameters after observing self-impingement of the emerging crystalline clusters due to periodic boundary conditions. In this section we investigate what happens to the quiescent systems at longer times, during which the degree of crystallinity (shown in Figure 17, for a single trajectory) reaches a quasi-plateau, as the self-impingement of the largest nucleus hinders growth.

**Figure 17:**
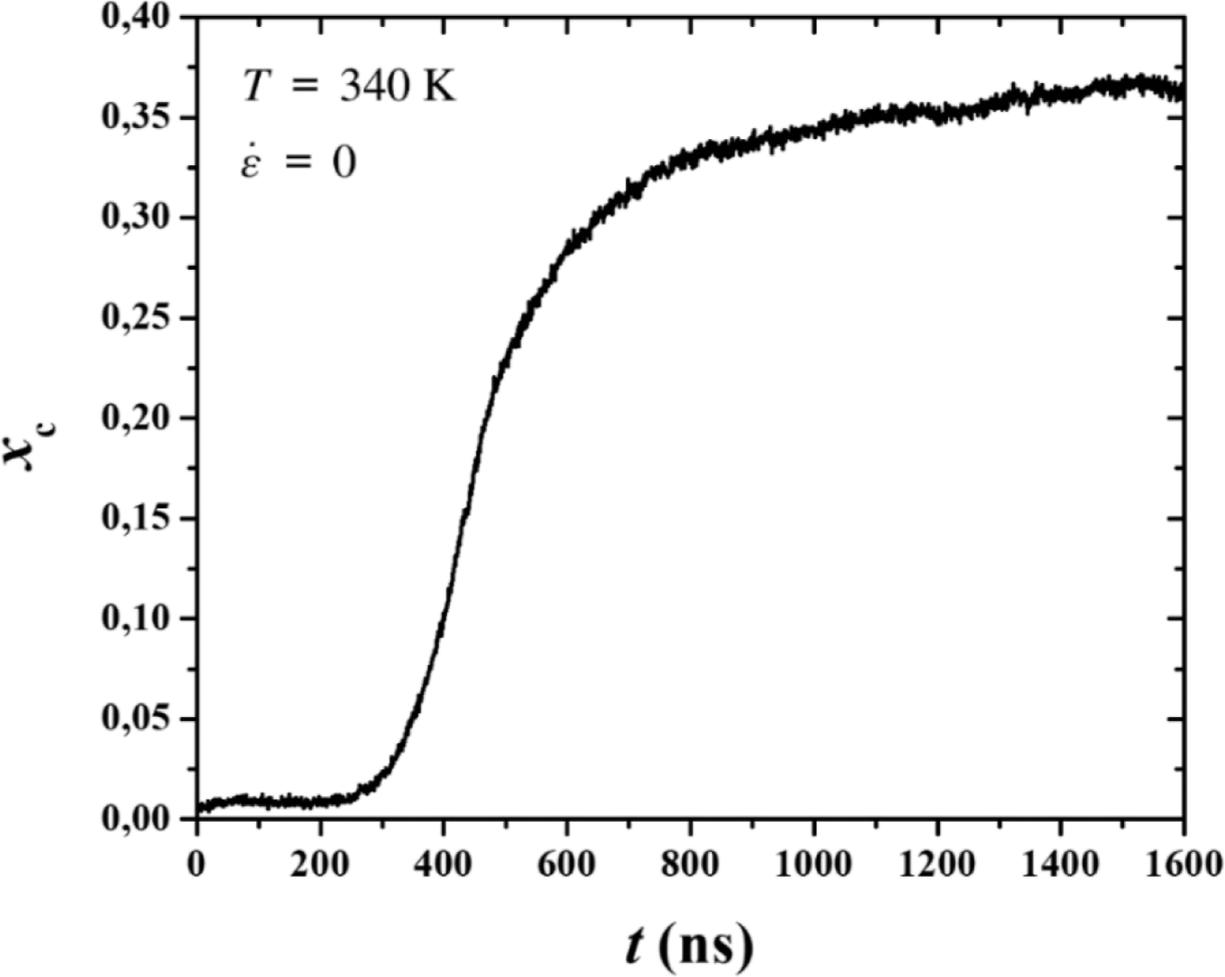
Degree of crystallinity, *x*_c_, as a function of time, for a single PE trajectory under quiescent conditions.

The picture of the morphology obtained under stretching conditions is very clear in this work, where we observe a highly cylindrical cluster symmetry, due to the very obvious mergers of similarly oriented different nuclei, which is also probably indicating the birth of a shish structure.^47^ It is harder to discern a terminal morphology in our quiescent simulations, however. Experimentally, PE crystallization under quiescent conditions leads to spherulitic structures.^72^ The radius of a spherulite in HDPE is reported as 30 to 150 μm,^73^ while our simulation box edge length is approximately 14 nm. Therefore, we can only hope to observe what happens within a very small region of material inside a spherulite. In some of our quiescent simulations, a second nucleus emerged during the time frame of observation. In Figure 18 we display a 3D representation of two discrete crystalline nuclei in a quiescent specimen at *t* = 1600 ns, hundreds of ns after nucleation (τ_i_* = 261 ns), where self-impingement of the largest nucleus with its periodic images has taken place, and the degree of crystallinity has reached a quasi-plateau. The second nucleus emerged spontaneously at approximately 149 ns after the first one, at *t* = 410 ns. Both nuclei exhibit similar quasi-spherical symmetry; the orientation of their respective crystalline strands is completely different, however, in accordance with the orientational randomness shown in Section 4.5.2. A solitary chain acting as a bridge can be noticed between them, the whole morphology being reminiscent of the computational findings of Verho et al.^31^ The two nuclei do not ultimately merge, which can be attributed to the different orientation of the crystalline strands, as well as to their significant confinement in the simulation box.

**Figure 18:**
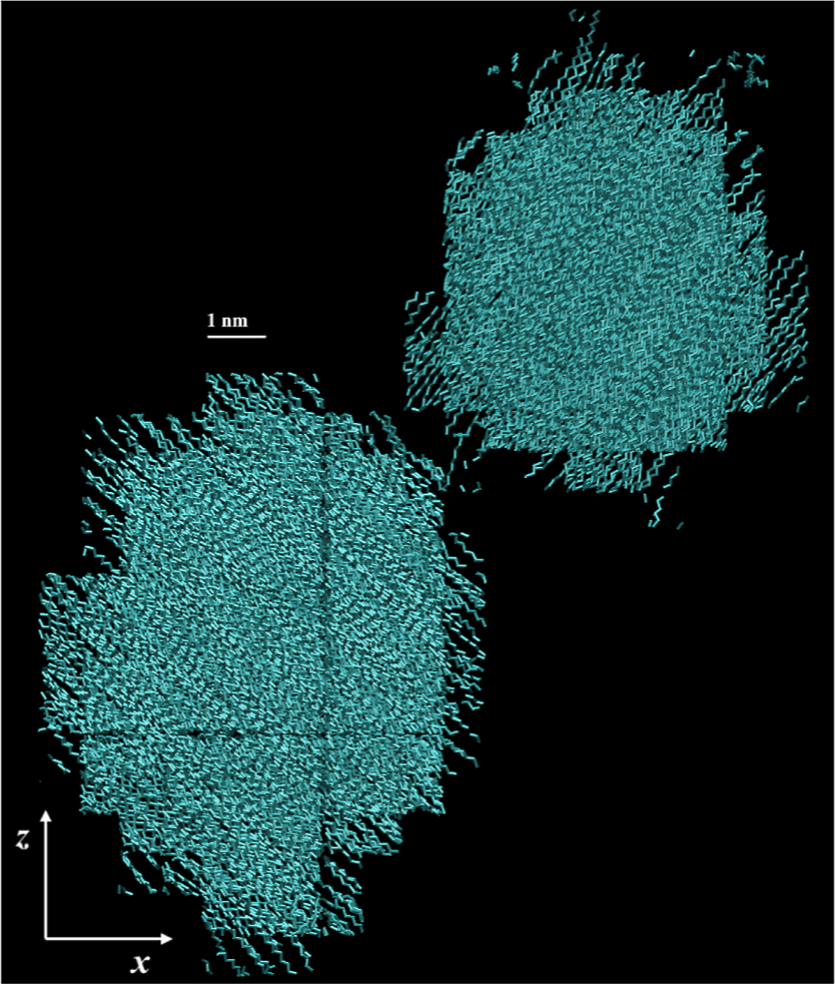
Snapshots of two crystalline nuclei in a single simulation run generated under quiescent conditions, at *t* = 1600 ns. The temperature is 340 K. The snapshots were created using VMD software.^60,61^

An important morphological feature observed in spherulites is curvature, a gradual turning of the lamellar plane.^72,74^ In Figure 19, we show a timelapse of a curved crystal being generated under quiescent conditions in one of our simulation runs. In a very rare instance among our specimens, a second nucleus appeared early, shortly after the first successful nucleation event; at the same time, the orientation of those two nuclei was similar within a narrow margin, but not identical, as displayed in Figure 19a. Shortly afterward, the two nuclei started merging via a shared narrow edge, as shown in Figure 19b. The slight difference in orientation resulted in the curved crystalline structure shown in Figure 19c, in which a second curved crystallite is also visible, below the original one. These observations could very well point toward one probable mechanism for the initiation of curvature in spherulites, linking local orientation with morphology. Unlike stretch-induced crystallization, there is no inherent nematic orientation under quiescent conditions, making merging events between different clusters much sparser, compared to the rapid merging under stretching which is showcased multiple times in this study. Regrettably, the current quiescent data is very limited, not allowing us to pinpoint an exact mechanism for the development of this morphology; the data can only serve as a strong indication. Unquestionably, examining a larger simulation box, with more space for nucleation and growth to occur, would greatly enhance the possibility not only for an abundance of nuclei to emerge under quiescent conditions, but also for these nuclei to interact and develop the unique morphology observed experimentally under quiescent conditions.

**Figure 19:**
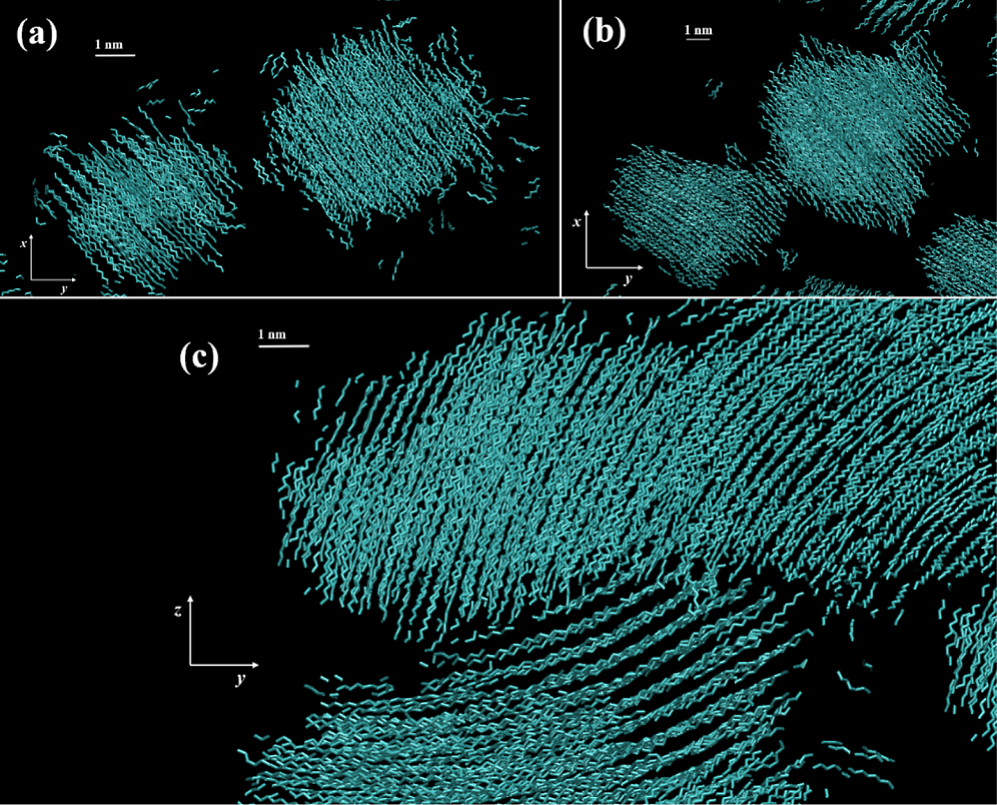
Snapshots of two crystalline nuclei in a single simulation run generated under quiescent conditions, at (a) *t*_1_ = 235 ns, (b) *t*_2_ = 250 ns and (c) *t*_3_ = 520 ns. The induction time of this system was determined at τ_i_* = 139 ns. The temperature is 340 K. The snapshots were created using VMD software.^60,61^

## 5 Conclusions

We have performed a thorough computational analysis of PE crystallization under quiescent and stretching conditions, focusing on a quantitative characterization of the morphology that develops as a function of time. By conducting MD simulations, starting from 24 different MC equilibrated and statistically independent initial configurations, we were able to work on a wide sample of data. We have calculated the degree of crystallinity across all systems studied. Through our home-built cluster analysis algorithm, we characterized the shape, size, and orientation of the developing ordered clusters. We have proposed and implemented a new method to estimate an individual induction time for each simulation trajectory by analyzing the temporal evolution of the radius of gyration of the largest ordered cluster encountered in it. Having determined those induction times, we have shown that they conform to a log-normal probability distribution, as seen experimentally. We also performed a kinetic analysis through an MFPT approach, based on the size (mass) of the largest cluster; through this we estimated the critical nucleus size, Zeldovich factor, induction time, nucleation rate and growth rate for both simulation protocols, comparing our findings to 3D snapshots of the systems at corresponding times. Results from our new radius of gyration-based method and from the MFPT method are fully consistent. Both nucleation and growth rates increase significantly in the presence of an external flow field, while the size of the critical nucleus is quite similar between the two protocols. Furthermore, we proceeded with a full shape and orientation analysis, based on diagonalizing the radius of gyration tensor and the chord-based **Q**-tensor of the largest ordered cluster in each system. From the radius of gyration tensor, we computed the squared radius of gyration of the cluster, as well as two very important shape measures, the reduced asphericity and acylindritity. The **Q**-tensor analysis, on the other hand, provided us with a scalar order parameter *S*_**Q**_ quantifying the degree of chord vector orientation and a director unit vector **n**_chords_ quantifying the prevalent local orientation of chords. Initially, ordered clusters were found to adopt roughly the same geometric features, regardless of the crystallization protocol (quiescent conditions or stretching). The presence of an elongational flow field, however, not only significantly accelerates nucleation, but also affects the morphology, by imposing strong orientation of the emerging crystalline regions along the drawing axis. This behavior, present even during the early stages of nucleation and fully established after nucleation and during growth, results in a fundamentally different morphology between the two cases. In the stretching case crystallites ultimately adopt a predominantly fractal-like cylindrical shape, elongated along the drawing direction, while the shape of the nuclei produced under quiescent conditions can be approximated by a sphere. The cylindrical shape observed in stretching simulations at long times stems mainly from clusters of chain stems oriented in the pulling direction merging together along that direction and can be proposed as a main contributor to the shish morphology.^47^ We have quantified the evolution of the shape of the largest crystallite by tracking the square roots of the three eigenvalues of its radius of gyration tensor, which are proportional to the lengths of the semiaxes of an ellipsoid representing the crystallite. From these we have extracted the growth rates along the principal axes. In the stretching case, mergers of crystallites at long times lead to a growth rate that is more than 3 times higher than the initial growth rate of a critical nucleus. We have determined a volumetric growth rate based on the clusters’ geometry. This volumetric growth rate was compared to the growth rates estimated from the MFPT analysis, finding very good coherence between the two methods. Finally, we provide evidence for the semicrystalline structure obtained at long simulation times from our quiescent specimens. We feature two distinct cases: (a) one where two crystallites of similar geometry but different nematic orientation do not merge, but bridging between them is observable, and (b) a second instance where we report merging between crystallites leading to a curved crystallite morphology, which could possibly be relevant to the curved lamellae at the center of a spherulite. Combining the information acquired by both our quiescent and stretch-induced crystallization simulations, we can conclude that the local orientation of crystalline strands comprising different crystalline clusters is key for merging between the clusters and for the type of morphology that can evolve. The methodology applied here can contribute to the development of a mesoscopic model of PE crystallization which will require fewer degrees of freedom and thus be less computationally intensive than atomistic simulations. Such a model would be capable of addressing longer length and time scales, as required in industrial production of semicrystalline polymeric materials. A systematic application over a range of temperatures, flow fields and molecular weight distributions can provide a wealth of data on nucleation and growth mechanisms and rates and on the development of semicrystalline morphologies. Expanding the novel methodologies presented in this work to a complete analysis not only of the largest cluster, but of all clusters present in the system, evaluating their size distribution, and quantifying important parameters, such as the cluster merging and demerging rates, as well as the explicit impact of smaller clusters on nucleation and growth, could provide key input for the development of a “bottom-up” mesoscopic model for flow-induced crystallization. Such a mesoscopic model could help design molecular architectures, molecular weight distributions, and processing conditions leading to semicrystalline polymer products with tailored mechanical and permeability properties.

## Supplementary Material















## Notes

S1. Extraction of characteristic times (entanglement relaxation time τ_e_, Rouse time, τ_R_ and disengagement (longest relaxation) time, τ_d_) in the melt. S2. Radius of gyration of PE chains. S3. Fits to the principal dimensions Λ_1_, Λ_2_, Λ_3_ of crystallites as functions of time used for the extraction of growth rates. S4. Relative changes in the different rooted eigenvalues. S5. Proportionality constant between ellipsoid volume and actual cluster volume (PDF)
S6. Video of a PE system undergoing quiescent crystallization (MP4)
S7. Video of the evolution of the largest crystalline cluster in a PE system undergoing quiescent crystallization (MP4)
S8. Video of a PE system undergoing stretch-induced crystallization (MP4)
S9. Video of the evolution of the largest crystalline cluster in a PE system undergoing stretch-induced crystallization (MP4)
S10. Video of a single PE chain in melt undergoing quiescent crystallization (MP4)
S11. Video of a single PE chain in melt undergoing stretch-induced crystallization (MP4)
