# Supplementary material for: A Computational Analysis of Crystallite Shape under Quiescent and Stretch-Induced Polyethylene Crystallization
Source: Macromolecules. 2025 Nov 30;58(23):12448–65. doi: 10.1021/acs.macromol.5c02153 (PMC12874634; doi:10.1021/acs.macromol.5c02153)
Supplement: Supplementary file 1 [file ma5c02153_si_001.pdf]

Supporting Information

A Computational Analysis of Crystallite Shape under Quiescent and Stretch-induced Polyethylene Crystallization

*Fotis Venetsanos, Stefanos D. Anogiannakis, Doros N. Theodorou**

School of Chemical Engineering, National Technical University of Athens, 9 Heroon Polytechniou Street, 15780 Athens, Greece

S1. Extraction of characteristic times (entanglement relaxation time τ_e, Rouse time, τ_R and disengagement (longest relaxation) time, τ_d) in the melt

We have extracted the characteristic times for our system by extrapolating from available data of previously conducted melt simulations in our lab, using the same model, for systems of monodisperse PE melts containing N_{ch} = 16 polymer chains, each consisting of N = 500 methylene atoms. We consider the differences between this monodisperse system and the C1000 system in this work negligible, due to the polydispersity index ($Đ$ = 1.08) of the latter being very close to unity. In Figure S1a, we display the segmental monomer mean square displacement (MSD),

$$g_1(t) = \frac{1}{N_{ch}} \frac{1}{N} \sum_{i=1}^{N_{ch}} \sum_{n=n_1}^{n_2} \left\langle \left[r_{i,n}(t) - r_{i,n}(t) \right]^2 \right\rangle \tag{S1}$$

To reduce chain end effects, the MSD was averaged over approximately the 10% of the central beads (from beads n_1 = 112 to n_2 = 138) of each chain. The corresponding τ_e and τ_R are calculated from changes in the slope of the g_1(t) plot in logarithmic coordinates and found to be 1.7 ns and 38 ns, respectively. In Figure S1b, we show the end-to-end autocorrelation function,

$$\Phi(t) = \left\langle \frac{\mathbf{R}_{ee}(t)}{\left|\mathbf{R}_{ee}(t)\right|} \cdot \frac{\mathbf{R}_{ee}(0)}{\left|\mathbf{R}_{ee}(0)\right|} \right\rangle \tag{S2}$$

for the C500 system, at T = 450 K. From this we estimate τ_d at 627.55 ns, by fitting a single exponential decay on $\Phi(t)$.

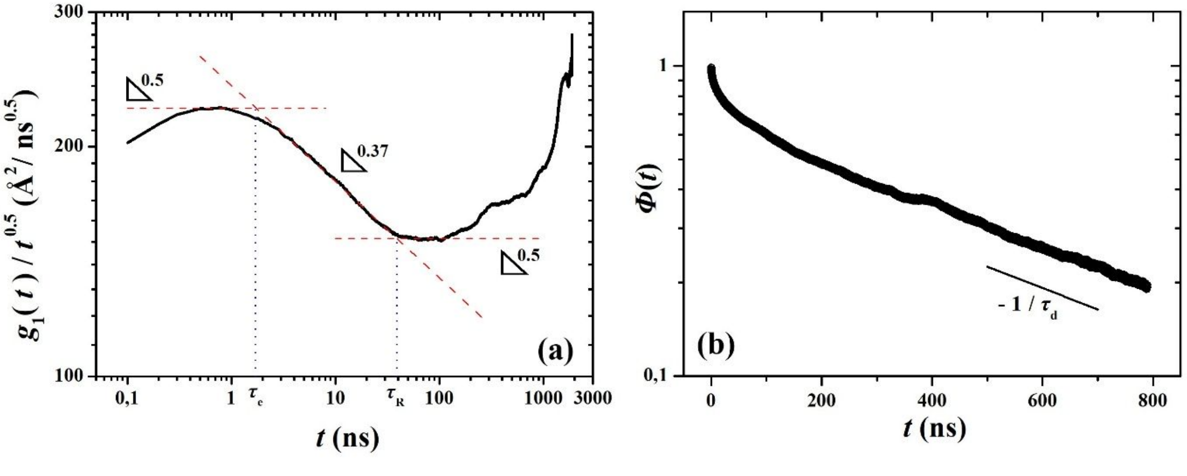

Figure S1. (a) Segmental monomer mean square displacement (MSD), g_1(t), and (b) end-to-end autocorrelation function, as functions of time, for the C500 PE system, at T = 450 K, highlighting our estimates (a) of the entanglement relaxation time, τ_e, and Rouse time, τ_R, and (b) of the disengagement time, τ_d.

From these values, we can extrapolate from the C500 to the C1000 system studied in our work, and from the melt temperature at 450 K to $T = 340$ K, the temperature in which we perform our crystallization simulations. The entanglement relaxation time has a very weak dependence on molecular weight. The Rouse time and the disengagement time are proportional to N^2 and $N^{3.4}$, respectively, N being the (average) number of monomers per chain in a system,[S1] therefore we can easily calculate the corresponding relaxation times for our system at 450 K. To finally calculate the Rouse time at the crystallization temperature, $T = 340$ K, we invoke a generic Williams-Landel-Ferry (WLF) equation,

$$\tau_R(T) = \tau_R(T_g)\exp\left(-C_1\frac{T-T_g}{T-T_g+C_2}\right), (T > T_g) \quad \text{(S3)}$$

with the universal parameters $C_1 = 17.44$ and $C_2 = 51.6$ K and T_g being the glass transition temperature of PE.[S2] The glass transition temperature chosen is $T_g = 150$ K.[S3] Equation S3 is similarly used for the extraction of both the entanglement time, τ_e, and disengagement time, τ_d, as well.

S2. Radius of gyration of PE chains

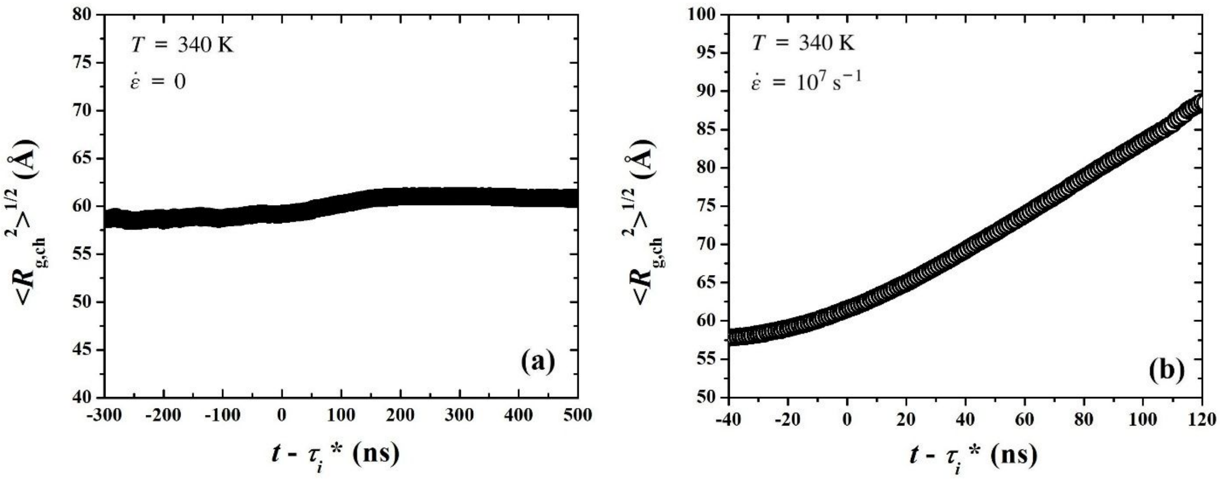

Figure S2. Average root mean square radius of gyration of the PE chains, averaged over all simulation runs, as a function of time shifted by τ^*, under (a) quiescent and (b) stretching conditions.

S3. Fits to the principal dimensions Λ_1, Λ_2, Λ_2 of crystallites as functions of time used for the extraction of growth rates

We modeled the time evolution of the square roots Λ_1, Λ_2, Λ_3 of the eigenvalues of the radius of gyration tensor **S** of the largest ordered cluster invoking a linear fit at the central part of the curve,

$$\Lambda_{\mathrm{i}} = a + G_{\mathrm{fit}}^{-1} t \tag{S4}$$

, a being the intercept, G_{fit} the slope and fitted growth rate and $i = 1$, 2 or 3 the index of the square root of the eigenvalue being examined. The values of the fitted parameters are shown in Table S1. The fits are displayed in Figure S3.

Table S1. Fit parameter values, subscript q and s denoting quiescent and stretch-induced crystallization, respectively.

Function	a (Å)	G_{fit} (Å/ns)
$\Lambda_{1,\mathrm{q}}$	9.3	0.11
$\Lambda_{2,\mathrm{q}}$	7.2	0.10
$\Lambda_{3,\mathrm{q}}$	5.7	0.09
$\Lambda_{1,\mathrm{s,1st\ region}}$	10.6	0.30
$\Lambda_{1,\mathrm{s,2nd\ region}}$	6.7	0.47
$\Lambda_{2,\mathrm{s}}$	6.9	0.28
$\Lambda_{3,\mathrm{s}}$	4.6	0.28

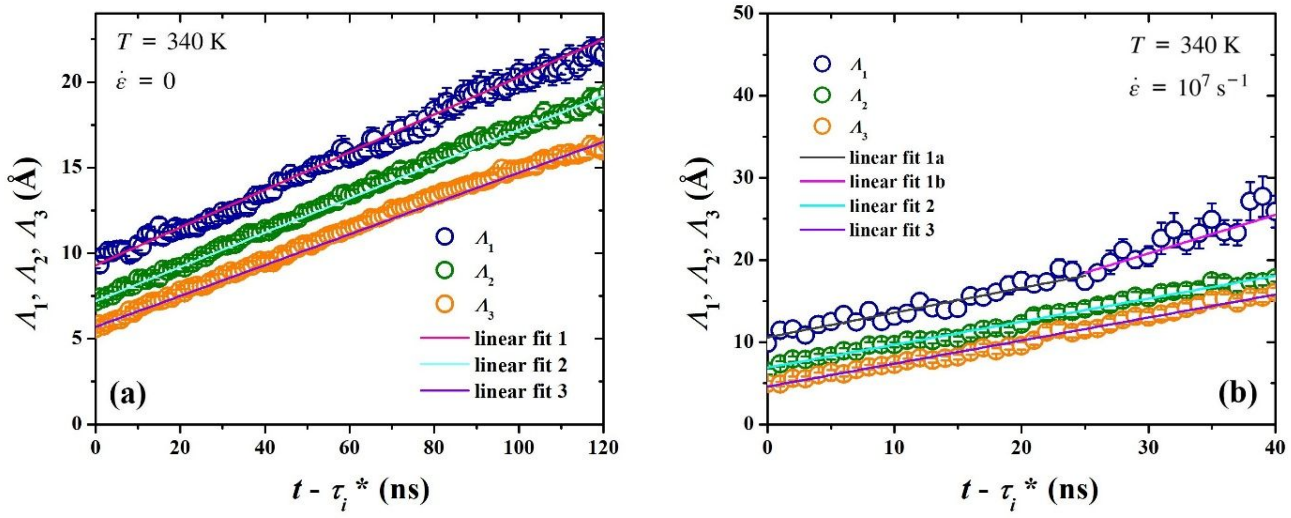

Figure S3. Linear fits on the square roots of the eigenvalues Λ_1, Λ_2, Λ_3 of the radius of gyration tensor S of the largest ordered cluster as functions of time relativeto the induction time under (a)

quiescent and (b) stretching conditions. The slope of the linear fits is proportional to the growth rate along each axis.

S4. Relative changes in the different rooted eigenvalues

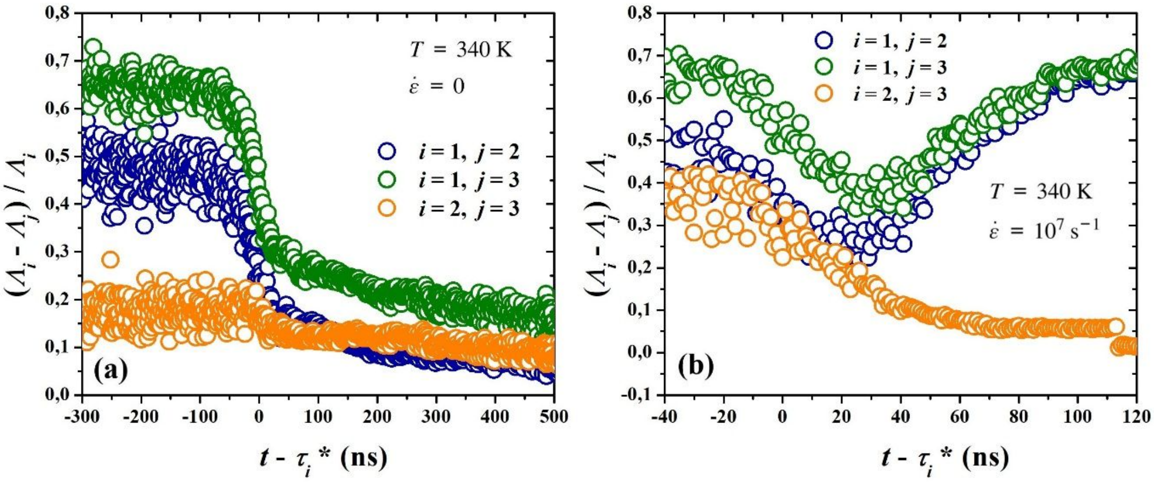

Figure S4. Reduced differences between pairs of the rooted eigenvalues $\Lambda_1>\Lambda_2>\Lambda_3$ of the radius of gyration tensor **S** of the largest ordered cluster as functions of time relative to the induction time under (a) quiescent and (b) stretching conditions.

S5. Volume proportionality constant

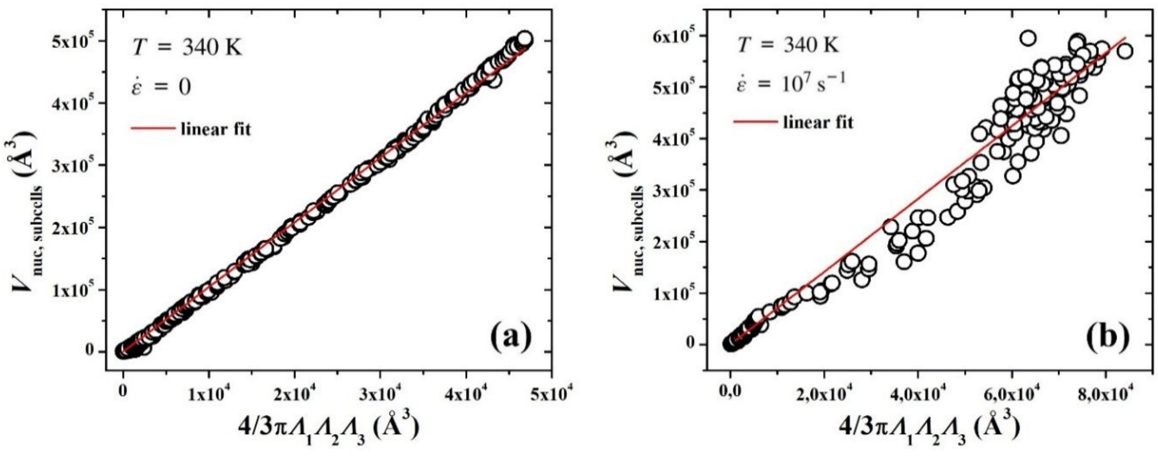

Figure S5. Volume of the largest crystalline cluster, as obtained by the TETRIS plots[S4], plotted against $\frac{4}{3}\pi\Lambda_1\Lambda_2\Lambda_3$, under (a) quiescent and (b) stretching conditions. The slope of the linear fit corresponds to the proportionality constant.

S6. Video of a PE system undergoing quiescent crystallization.

'SV1_quiescent_system.mp4': In this video we display a PE system consisting of 100 chains with an average chain length of 1,000 methylene units, undergoing quiescent crystallization at constant temperature $T = 340$ K. The time step between two successive frames is $\Delta t = 10$ ns. Pink and cyan segments correspond to the amorphous and crystalline regions, respectively. The video was created using VMD software.[S5,S6]

S7. Video of the evolution of the largest crystalline cluster in a PE system undergoing quiescent crystallization.

'SV2_quiescent_largest_nucleus.mp4': In this video we display the evolution of the largest crystalline cluster for a PE system consisting of 100 chains with an average chain length of 1,000 methylene units, undergoing quiescent crystallization at constant temperature $T = 340$ K. The time step between two successive frames is $\Delta t = 10$ ns. We can discern that the identity of the largest nucleus changes during the incubation period, until a few nanoseconds prior to the creation of a stable nucleus. The roughly spherical shape of the cluster can be clearly identified. The simulation run is the same as in SV1_quiescent_system.mp4. The video was created using VMD software.[S5,S6]

S8. Video of a PE system undergoing stretch-induced crystallization.

'SV3_stretching_system.mp4': In this video we display a PE system consisting of 100 chains with an average chain length of 1,000 methylene units, undergoing crystallization under uniaxial stretching, along the *x*-axis, at constant temperature $T = 340$ K and under constant strain rate &= $10^{7}\,\mathrm{s}^{-1}$, from $\lambda = 1.0$ up to $\lambda = 2.0$. The time step between two successive frames is $\Delta t = 5$ ns. Pink and cyan segments correspond to the amorphous and crystalline regions, respectively. The video was created using VMD software.[S5,S6]

S9. Video of the evolution of the largest crystalline cluster in a PE system undergoing stretch-induced crystallization.

'SV4_stretching_largest_nucleus.mp4': In this video we display the evolution of the largest ordered cluster in a PE system consisting of 100 chains with an average chain length of 1,000 methylene units, undergoing crystallization under uniaxial stretching, along the *x*-axis, at constant

temperature $T = 340$ K and under constant strain rate $\& = 10^{7}\,\mathrm{s}^{-1}$, from $\lambda = 1.0$ up to $\lambda = 2.0$. The time step between two successive frames is $\Delta t = 5$ ns. Again, the largest nucleus changes its identity during the incubation period. Notably, under stretching, the largest cluster can change its identity post nucleation as well. As multiple crystalline clusters of similar size emerge (as opposed to the singular sizeable cluster appearing under quiescent conditions), one can take the place of another as the largest cluster in the system. Finally, the merging between the different clusters is prominently shown during the last frames of the video. The simulation run is the same as in SV3_stretching_system.mp4. The video was created using VMD software.[S5,S6]

S10. Video of a single PE chain undergoing quiescent crystallization.

'SV5_quiescent_chain.mp4': In this video we display how a single PE chain belonging to a PE system consisting of 100 chains with an average chain length of 1,000 methylene units, undergoing quiescent crystallization at constant temperature $T = 340$ K. The time step between two successive frames is $\Delta t = 5$ ns. Chain folding can be observed after nucleation. The video was created using VMD software.[S5,S6]

S11. Video of a single PE chain undergoing stretch-induced crystallization.

'SV5_quiescent_chain.mp4': In this video we display how a single PE chain belonging to a PE system consisting of 100 chains with an average chain length of 1,000 methylene units, undergoing crystallization under uniaxial stretching, along the *x*-axis, at constant temperature $T = 340$ K and under constant strain rate $\& = 10^{7}\,\mathrm{s}^{-1}$, from $\lambda = 1.0$ up to $\lambda = 1.8$. The result is a chain heavily elongated, with strongly ordered segments along the deformation axis. Folding is observed in this case as well. The video was created using VMD software.[S5,S6]

REFERENCES

(S1) Rubinstein, M.; Colby, R. H. *Polymer Physics*; Oxford University, **2003**, 255-257.

(S2) Ziabicki, A. Crystallization of polymers in variable external conditions. *Colloid Polym. Sci.* **1996**, *274*, 705–716.

(S3) Theodorou, D. N. Simple model for the fracture of a polymer chain: Single-bond potential of mean force and tension-based rate constants for chain rupture. *J. Chem. Phys.* **2024**, *161*, 184905.

(S4) Anogiannakis, S. D.; Venetsanos, F.; Theodorou, D. N. Simulating Stretch-Induced Crystallization of Polyethylene Films: Strain Rate and Temperature Effect on the Kinetics and Morphology. *Macromolecules* **2024**, *57*, 7331–7346.

(S5) Humphrey, W.; Dalke, A.; Schulten, K. VMD - Visual Molecular Dynamics. *J. Mol. Graphics* **1996**, *14*, 33–38.

(S6) http://www.ks.uiuc.edu/Research/vmd/
